# Large‐eddy simulation of foehn–cold pool interactions in the Inn Valley during PIANO IOP 2

**DOI:** 10.1002/qj.3954

**Published:** 2021-01-17

**Authors:** L. Umek, A. Gohm, M. Haid, H. C. Ward, M. W. Rotach

**Affiliations:** ^1^ Department of Atmospheric and Cryospheric Sciences University of Innsbruck Innsbruck Austria

**Keywords:** complex terrain, foehn, cold‐air pool, heat budget, large‐eddy simulation, shear flow instability, turbulent erosion

## Abstract

Processes of cold‐air pool (CAP) erosion in an Alpine valley during south foehn are investigated based on a real‐case large‐eddy simulation (LES). The event occurred during the second Intensive Observation Period (IOP 2) of the PIANO field experiment in the Inn Valley, Austria, near the city of Innsbruck. The goal is to clarify the role of advective versus turbulent heating, the latter often being misrepresented in mesoscale models. It was found that the LES of the first day of IOP 2 outperforms a mesoscale simulation, is not yet perfect, but is able to reproduce the CAP evolution and structure observed on the second day of IOP 2. The CAP exhibits strong heterogeneity in the along‐valley direction. It is weaker in the east than in the west of the city with a local depression above the city. This heterogeneity results from different relative contributions and magnitudes of turbulent and advective heating/cooling, which mostly act against each other. Turbulent heating is important for faster CAP erosion in the east and advective cooling is important for CAP maintenance to the west of Innsbruck. The spatial heterogeneity in turbulent erosion is linked to splitting of the foehn into two branches at the mountain range north of the city, with a stronger eastward deflected branch. Intensification of the western branch at a later stage leads to complete CAP erosion also to the west of Innsbruck. Above the city centre, turbulent heating is strongest, and so is advective cooling by enhanced pre‐foehn westerlies. These local winds are the result of CAP heterogeneity and gravity‐wave asymmetry. This study emphasizes the importance of shear‐flow instability for CAP erosion. It also highlights the large magnitudes of advective and turbulent heating compared to their net effect, which is even more pronounced for individual spatial components.

## Graphical abstract

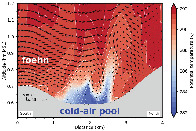

Turbulent erosion of a cold‐air pool in the Austrian Inn Valley during foehn is investigated based on a large‐eddy simulation and a comprehensive heat budget analysis. It is found that cold‐pool erosion is governed by a complicated interplay between turbulent and advective heating/cooling that is strongly variable in time and space. The figure illustrates turbulent cold‐pool erosion by shear‐flow instability induced by the foehn flow.

## 1 INTRODUCTION

Mountains have a profound impact on the atmosphere. For example, they modify synoptic‐scale flow and influence boundary‐layer processes and turbulence (e.g., Whiteman, 2000). Hence, their influence on atmospheric processes ranges from 𝒪(10^2^) to 𝒪(10^6^) m and from seconds to a few days (Serafin *et al*., 2018). Among the mountain‐induced phenomena, downslope windstorms can be observed in mountainous regions around the globe and bear different local names such as foehn in the European Alps. Due to the severity of these winds on the leeward side of mountains (Durran, 1990), downslope windstorms like Alpine foehn can cause damage to infrastructure and pose a hazard to aviation (e.g., Gohm *et al*., 2008; Chan and Hon, 2016). Additionally, the concentration of near‐surface pollutants in valleys can be either decreased or increased by the breakthrough of foehn to the valley bottom (e.g., Seibert *et al*., 2000; Gohm *et al*., 2009; Harnisch *et al*., 2009). Therefore, a proper forecasting of foehn events by numerical weather prediction (NWP) models is desirable. However, mesoscale NWP models partly fail in realistically representing foehn in Alpine valleys due to a coarse grid spacing and insufficient representation of local topography and turbulent processes (Gohm *et al*., 2004; Zängl *et al*., 2004b; Sandner, 2020).

**FIGURE 1:**
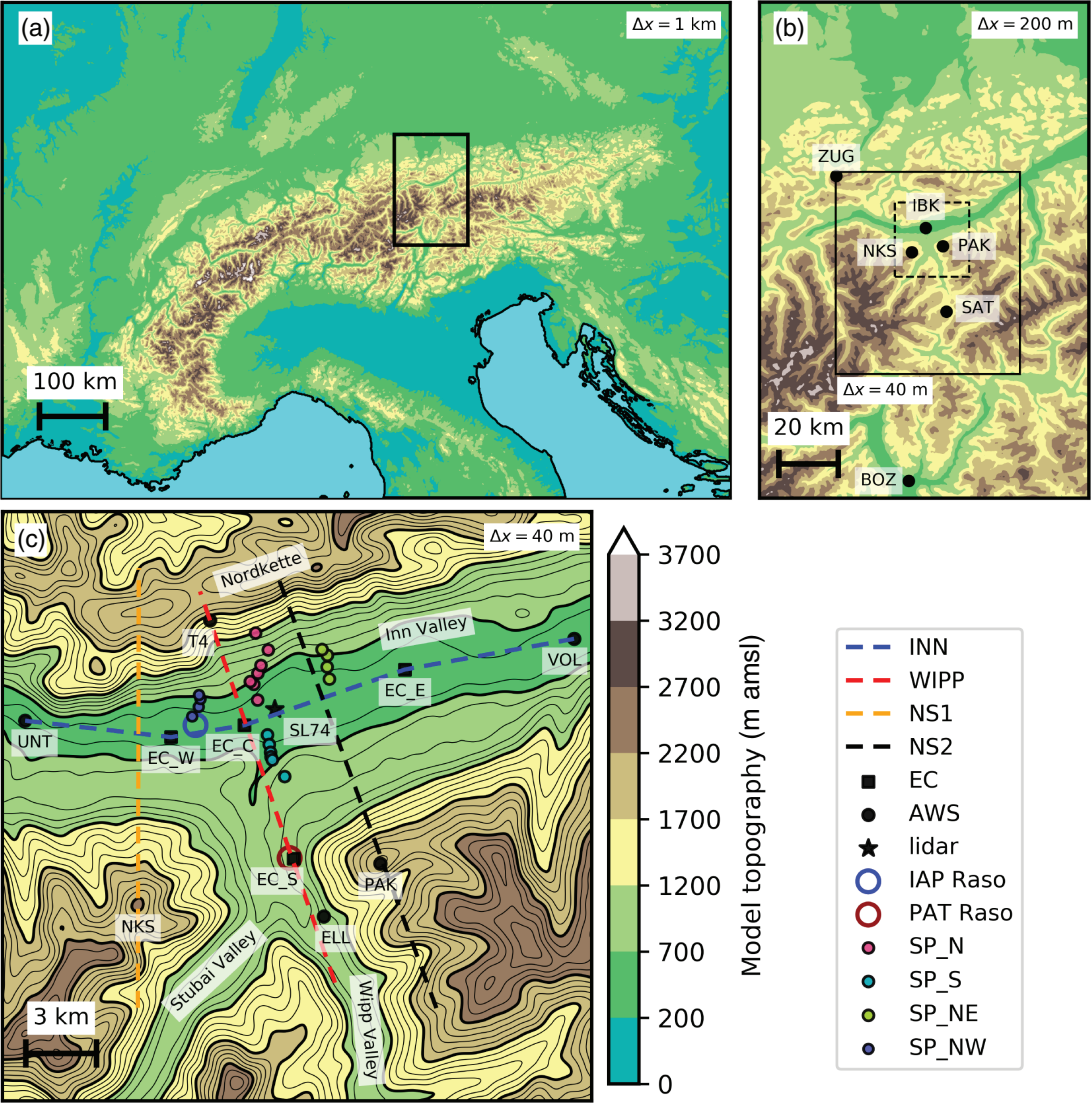
WRF model topography (colour contours, 500 m interval) together with landmarks and locations of instruments mentioned in the text. (a) Mesoscale WRF domain (MESO) with Δx = 1 km covering the greater Alpine region. The extent of the outer LES domain with Δx=200 m (LES‐dx200) is indicated by the black rectangle. (b) Domain of LES‐dx200. The black rectangle outlines the nested inner LES domain with Δx=40 m (LES‐dx40) while the dashed rectangle indicates the subdomain shown in (c). Locations of Bozen (BOZ), Sattelberg (SAT), Patscherkofel (PAK), Nockspitze (NKS), Innsbruck (IBK) and Zugspitze (ZUG) are shown by black dots. (c) Subdomain of LES‐dx40 around IBK. Thick (thin) black contour lines represent the LES model topography with an interval of 500 (100) m. Black dots and squares indicate locations of automatic weather (AWS) and eddy covariance (EC) stations, respectively. The black star denotes the position of a lidar on a high building in the city of Innsbruck (SL74). Coloured open circles indicate launch sites of radiosondes from Innsbruck airport (IAP Raso) and near the village of Patsch (PAT Raso). Filled circles show stations of the four slope profiles SP_N, SP_NW, SP_NE and SP_S. Dashed coloured lines denote the orientation of of four vertical cross‐sections along the Inn Valley (INN), the Wipp Valley (WIPP), and across the Inn Valley west (NS1) and east (NS2) of Innsbruck

Foehn in the Alpine region and around Innsbruck (IBK, Figure 1b) has been the subject of a wide range of scientific studies, many of them conducted during the Mesoscale Alpine Programme (MAP; Bougeault *et al*., 2001). The city of Innsbruck is located at about 570 m above mean sea level (amsl), where the south–north aligned Wipp Valley meets almost perpendicularly with the west–east orientated Inn Valley (Figure 1b,c). The Wipp Valley is very prone to foehn, as it connects to the lowest pass of the Alps, the Brenner pass, at 1,371 m amsl. The pass region and the nearby mountain Sattelberg (2,107 m amsl; SAT in Figure 1b) form a *gap* in the main Alpine crest which promotes the occurrence of south foehn downstream in the Wipp Valley (Mayr *et al*., 2007). South foehn in the Wipp Valley has been explained as a response to a temperature difference between the air masses north and south of the main Alpine crest (Armi and Mayr, 2007; Mayr and Armi, 2008) and may be distinguished into a shallow and a deep type (Mayr *et al*., 2007). Shallow foehn is characterized by southerly winds near SAT and in the Wipp Valley up to crest height, while more westerly winds (i.e., roughly parallel to the main Alpine ridge) prevail above. Therefore, shallow foehn is considered as a *gap flow* (Gohm and Mayr, 2004; Mayr *et al*., 2004; 2007; Zängl *et al*., 2004a). In contrast, deep foehn features southwesterly to southerly (cross‐Alpine) large‐scale flow above crest height.

In a geographical setting like the Inn/Wipp Valley intersection, a cold‐air pool (CAP) induced by nocturnal cooling is often present in the Inn Valley, while foehn has already established within the Wipp Valley (Mayr *et al*., 2007). A foehn breakthrough in the downwind Inn Valley subsequently only occurs if this CAP is removed. NWP models often struggle with the formation of such a CAP in complex terrain due to deficiencies in the representation of the topography, land surface–atmosphere interactions or limitations in resolving boundary‐layer processes (Lareau *et al*., 2013). However, since the CAP determines the near‐surface stratification in the valley, it also determines the time of the foehn breakthrough at the valley bottom. Therefore, the correct formation of CAPs in NWP models, the interaction between the CAP and the overlying foehn flow, and finally the CAP erosion are crucial for an accurate forecast of foehn breakthrough.

The CAP erosion can be caused by a variety of atmospheric processes which are described in detail in Haid *et al*. (2020) and repeated here for clarity.

(a) Bottom‐up heating in the CAP by surface sensible heat flux convergence associated with short‐wave incoming radiation (e.g., Gubser and Richner, 2001; Mayr and Armi, 2010),
(b) Top‐down erosion of the CAP by Kelvin–Helmholtz (K‐H) instabilities (e.g., Fritts 1982) at the foehn–CAP interface and microscale turbulent mixing inside the CAP (e.g., Nater *et al*., 1979; Zhong *et al*., 2003; Jaubert *et al*., 2005; Marić and Durran, 2009; Fritts *et al*., 2010; Lareau and Horel, 2015b; Tollinger *et al*., 2019), and
(c) Displacement of the CAP by the foehn flow (e.g., Flamant *et al*., 2006; Lareau and Horel, 2015a).

Haid *et al*. (2020) stress that there is disagreement in the literature on the relative importance of the various processes influencing the CAP removal. This is especially valid for mechanism (b), partly due to limitations in explicitly resolving this process in both observations and simulations. Moreover, the role of horizontal versus vertical turbulent mixing has not been investigated thoroughly in the past.

The most frequent wind direction in Innsbruck during south foehn is south (called direct foehn) with foehn winds emanating from the Wipp Valley (Plavcan, 2014). However, the foehn flow can also be deflected by the mountain range north of Innsbruck (Nordkette; Figure 1c). This flow deflection can lead to variable low‐level wind directions in and around Innsbruck (Zängl *et al*., 2004b; Plavcan, 2014; Haid *et al*., 2020). Prior to foehn breakthrough in the Inn Valley, *pre‐foehn westerlies* are commonly observed in the CAP (Seibert, 1985; Zängl, 2003; Zängl and Gohm, 2006; Muschinski *et al*., 2020). Pre‐foehn westerly wind speed generally exceeds the strength of thermally driven down‐valley winds. The pre‐foehn westerlies are hypothesized to be caused by an asymmetry in the gravity‐wave structure east and west of the Wipp Valley exit (Zängl, 2003) or an asymmetry in the CAP depth with a shallower CAP east of Innsbruck (Zängl and Gohm, 2006). Both processes enhance the along‐valley pressure gradient. Muschinski *et al*. (2020) found a nearly linear relation between this pressure gradient and the along‐valley CAP heterogeneity for three out of six foehn events.

While prior observational and numerical studies mostly focused on the synoptic‐scale forcing and the well‐developed phase of the foehn, this study draws scientific attention to the turbulent aspects of foehn and especially the transient phase of the foehn breakthrough to the valley floor. In autumn and early winter 2017, a measurement campaign was conducted in the Inn and Wipp Valley as part of the research project Penetration and Interruption of Alpine Foehn (PIANO; e.g., Haid *et al*., 2020; Muschinski *et al*., 2020). A first detailed analysis based on observations collected during the second Intensive Observation Period (IOP 2) of the PIANO campaign on 4 and 5 November 2017, has already been conducted by Haid *et al*. (2020). This work also focuses on IOP 2 and complements the study of Haid *et al*. (2020) with mesoscale and large‐eddy simulations (LES) using the Weather Research and Forecasting (WRF) model. Tollinger *et al*. (2019) showed that mesoscale WRF simulations are able to partly resolve shear‐flow instabilities on the top of a downslope windstorm. However, a substantial decrease in the horizontal grid spacing is needed to fully resolve breaking K‐H waves and the associated turbulent mixing, which is achieved by conducting an LES in the present study. The LES of foehn in such a complex environment is unprecedented and together with turbulence‐resolving measurements gathered during the PIANO IOP 2 enables new insights into turbulent foehn–CAP interaction. More specifically, the LES enables the gap to be closed in previous CAP heat budget analyses based on observations due to missing or roughly estimated heating terms (e.g., Haid *et al*., 2020)

With the numerical simulation of PIANO IOP 2 and the rich observational dataset at hand, this study aims at answering the following scientific questions:

- Is the LES superior to a mesoscale simulation in predicting the foehn penetration to the valley bottom?
- Are turbulent processes of foehn–CAP interaction and CAP erosion sufficiently resolved in the LES?
- Can the LES reproduce the observed spatial CAP heterogeneity in the Inn Valley and what are the main processes responsible for this heterogeneity?
- What is the relative importance of different processes contributing to the removal of the Inn Valley CAP in the LES, such as mean‐flow temperature advection, resolved and subgrid‐scale (SGS) turbulent mixing, as well as microphysical and radiative processes?

The study is organized as follows. In Section 2 the PIANO field campaign, the WRF model set‐up and model diagnostics are introduced. Section 3 includes a brief summary of the synoptic situation during IOP 2 (Section 3.1) and results from the mesoscale WRF simulation (Section 3.2). Different characteristics of the LES are presented in Section 4 while Section 5 focuses on the CAP heat budget in the LES. Results are discussed in Section 6 and conclusions are drawn in Section 7.

## 2 DATA AND METHODS

### 2.1 PIANO field campaign

The field campaign of the PIANO research project took place in autumn and early winter 2017 around the city of Innsbruck, Austria (IBK in Figure 1b). The field campaign was characterized by seven Intensive Observation Periods (IOPs) consisting of six south foehn events and one west foehn event. A dense and diverse array of measurement systems was installed in and around Innsbruck during the PIANO field campaign in addition to routine observations by automatic weather stations (AWSs) in the area. Within this section, a non‐exhaustive overview of the PIANO instrumentation is given and the reader is referred to Haid *et al*. (2020) and Muschinski *et al*. (2020) for more detail on individual instruments.

Locations of different instruments are shown in Figure 1b, c. Routine AWSs at elevated sites (indicated by black dots in Figure 1b,c, respectively) include Zugspitze (ZUG; 2,967 m amsl), operated by the German Weather Service (DWD), Patscherkofel (PAT; 2,251 m amsl) and a slope station north of Innsbruck (T4; 1,566 m amsl). The latter two are operated by the Austrian Weather Service (ZAMG). Moreover, the Department of Atmospheric and Cryospheric Sciences of the University of Innsbruck (ACINN) routinely operates an AWS at Sattelberg (SAT; 2,107 m amsl) near the main Alpine crest and at Ellboegen (ELL; 1,080 m amsl) in the Wipp Valley. Additionally, data are used from an AWS at Bolzano in Italy (BOZ; 262 m amsl) operated by the Weather Service of the Autonomous Province of South Tyrol.

A total of nine portable AWSs were operated during the PIANO field campaign to increase the number of observations near the city of Innsbruck. These include the stations Unterperfuss (UNT; 594 m amsl) and Volders (VOL; 551 m amsl) indicated in Figure 1c, among others (Haid *et al*., 2020). Moreover, four eddy‐covariance stations (EC; squares in Figure 1c) were operated to the west (EC_W; 579 m amsl), near the city centre (EC_C; 621 m amsl), and to the east of Innsbruck (EC_E; 562 m amsl), as well as near the Wipp Valley exit (EC_S; 977 m amsl). EC_C was installed on the rooftop of ACINN at around 43 m above street level (Karl *et al*., 2020) while the sensors of the other three EC stations were located at about 2.5 m agl (above ground level). The EC stations sampled data at 20 Hz to derive turbulent fluxes, except for EC_C which sampled at 10 Hz. Radio soundings were performed near the village of Patsch (PAT; 962 m amsl) close to EC_S, as well as from Innsbruck Airport (IAP; 578 m amsl) near EC_W at a three‐hourly interval during individual IOPs (circles in Figure 1c). Within the city of Innsbruck, four scanning Doppler wind lidars were operated on top of high buildings. In this study data of the SL74 lidar on the PEMA building (Figure 1c) is used to derive the vertical velocity variance. The scanning strategy and further details regarding the lidar measurements are given in Haid *et al*. (2020).

To observe the spatial heterogeneity and vertical stratification of the CAP in the Inn Valley and the CAP erosion, temperature and relative humidity (T‐RH) sensors were distributed in and around the city. Within this study, data of four arrays of these instruments, installed along the sloping terrain north and south of Innsbruck, are used (SP_NW, SP_N, SP_NE, and SP_S in Figure 1c). Muschinski *et al*. (2020) describe the instruments in more detail and Haid *et al*. (2020) show that data from the temperature sensors are in good agreement with data gathered by radiosondes. Hence, data from these four slope profiles can be used to approximate the true vertical temperature profiles in the valley. To calculate potential temperature from air temperature observations by the T‐RH sensors, surface pressure measurements from the EC station closest to the corresponding slope profile are used. More specifically, pressure measured at the valley bottom is hydrostatically reduced to the location of the temperature observation on the slope by taking into account the height difference and the temperature gradient between the respective EC station and the T‐RH sensor. Pressure data from EC_C are used for SP_N and SP_S while data from EC_W (EC_E) are used for SP_NW (SP_NE). The derived potential temperature is then compared to WRF model data from the lowest model level at 10 m agl. Absolute height relative to msl between the observations and this model level differs by less than 14 m, except for the following stations of SP_S. Three sensors were installed on and near a ski jump at 666, 723 and 786 m amsl. Here, WRF data from model levels two, four and six at 668, 729, and 786 m amsl (30, 71, and 114 m agl) are used for comparison, respectively. The fourth sensor was installed on a flag pole at 878 m amsl. Here, data from WRF model level two at 877 m amsl (30 m agl) is used for the comparison.

### 2.2 Set‐up of the numerical model

The Advanced Research WRF model (ARW; version 4.1, Skamarock *et al*., 2019) is used in this study. The simulation strategy is twofold: A mesoscale WRF simulation (MESO) is conducted first and its output is subsequently used as initial and boundary conditions for a separate WRF LES. The LES aims at partly resolving the turbulent flow field in the target area. The numerical simulations performed in this study are briefly summarized in Table 1.

The MESO set‐up uses a single domain and a horizontal grid spacing of Δx=1 km, covering the whole Alpine region to capture the large‐scale flow (1,100 × 750 grid points; Figure 1a). The geographical extent and horizontal grid spacing are similar to the operational high‐resolution COSMO‐1 NWP model of MeteoSwiss. Initial and boundary conditions for MESO are provided by the operational high‐resolution (HRES) analysis of the European Centre for Medium‐Range Weather Forecasts (ECMWF) on model levels with a 0.1 × 0.1° latitude/longitude grid spacing and a 6‐hourly interval. The ECMWF analysis provides snow cover data, which is manually adjusted by removing snow below 1,600 m amsl. This height has been determined using webcam photographs taken during IOP 2. Furthermore, a user‐specified lapse‐rate for temperature extrapolation where the WRF model topography is below the lowest ECMWF model level is introduced in the WRF source code (Appendix B).

Output data of the mesoscale simulation at a 30‐min interval are used to generate initial and boundary conditions for a stand‐alone LES (using the ndown‐tool of the WRF software framework). The LES comprises two one‐way nested domains (Table 1 and Figure 1b) with a horizontal grid spacing of Δx=200 m (LES‐dx200) and Δx=40 m (LES‐dx40) with 405 × 735 and 1,150 × 1,500 grid points, respectively. Direct nesting of the LES into the mesoscale simulation proved unsuccessful due to numerical instabilities. The MESO simulations and the LES use the hybrid sigma‐pressure coordinate (Park *et al*., 2019) and a common set of 80 vertical mass levels with the first mass level at approximately 10 m agl. The vertical model level spacing is Δz=20 m near the surface and linearly stretched with increasing height up to a maximum value of Δz=400 m at the model top at around 19 km amsl. The uppermost 7 km act as a damping layer after Klemp *et al*. (2008) to avoid spurious wave reflections of vertically propagating gravity waves. The integration time step is 2.5 s for the MESO and 0.75 s and 0.25 s for LES‐dx200 and LES‐dx40, respectively.

The model orography of all domains is based on the Shuttle Radar Topography Mission digital elevation model (SRTM; USGS, 2000) with a horizontal grid spacing of 30 m. In order to avoid numerical instabilities caused by too steep terrain‐following coordinate surfaces, it is necessary to adjust the terrain of the LES. The model orography of LES‐dx200 is locally smoothed where slope angles exceed a threshold of 42°. This procedure mainly affects the complex orography north of the Nordkette and southwest of the Wipp and Stubai Valleys where elevations exceed 2,200 m amsl (Figure 1b,c). The topography of valley floors and moderately complex terrain is not or only marginally affected by this modification. LES‐dx40 uses the same orography as its parent domain, that is, the topography is interpolated from the 200 m grid to the 40 m grid to avoid numerical instabilities in LES‐dx40.

Furthermore, the WRF Pre‐Processing System (WPS) default soil and land‐use datasets are replaced by more current and detailed data. The Harmonized World Soil Database (HWSD; Milovac *et al*., 2014) with a horizontal mesh size of 30 arc‐seconds is used, as well as data from the CORINE Land Cover Inventory 2012 (European Environment Agency, 2017) with a horizontal mesh size of 100 m. CORINE land‐use data are reclassified to USGS land‐use classes following the approach of Pineda *et al*. (2004) with the addition of inland water bodies and three urban land‐use classes. Schmidli *et al*. (2018) showed that the use of high‐resolution, up‐to‐date land‐use and soil data improve numerical simulations in complex terrain.

All simulations use the Thompson microphysics parametrization (Thompson *et al*., 2008), the RRTMG short‐ and long‐wave radiation parametrizations (Iacono *et al*., 2008), the NOAH‐MP land surface model (Niu *et al*., 2011; Yang *et al*., 2011) and the revised MM5 surface layer parametrization (Jiménez *et al*., 2012). The latter is used due to its availability in both set‐ups of MESO and LES. Furthermore, topographic shading, slope effects on radiation, as well as horizontal diffusion in the physical space, are activated. The MESO set‐up uses the Mellor–Yamada–Nakanishi–Niino (MYNN) level 3 planetary boundary layer (PBL) parametrization (Nakanishi and Niino, 2006) for the vertical mixing and a first‐order closure after Smagorinsky (1963) for horizontal diffusion. In contrast, the LES uses the three‐dimensional SGS turbulence parametrization based on Deardorff (1980). The latter includes a prognostic equation for SGS turbulence kinetic energy (TKE). TKE is used to compute eddy viscosity and diffusivity for SGS turbulent mixing. The Deardorff constant (namelist variable c_k) is decreased from the default value of 0.15 to 0.09 similar to Lilly (1966, equation 51). Additionally, the length‐scale *ℓ* used in the calculation of the eddy viscosity and diffusivity is adjusted following Schmidli (2013, Appendix B).

To ensure numerical stability, a number of modifications to the default values of WRF namelist variables are essential. The coefficient epssm for time off‐centering in the vertically implicit time differencing scheme is increased from the default value of 0.1 to 0.5 (0.9) in the MESO (LES) set‐up. This increases dampening of instabilities associated with sound waves and sloping model levels (e.g., Dudhia, 1995). For LES‐dx40, the coefficients for divergence damping smdiv and external mode damping emdiv are increased from 0.1 to 0.2 and from 0.01 to 0.02, respectively.

### 2.3 Generating initial conditions for the LES

**FIGURE A1:**
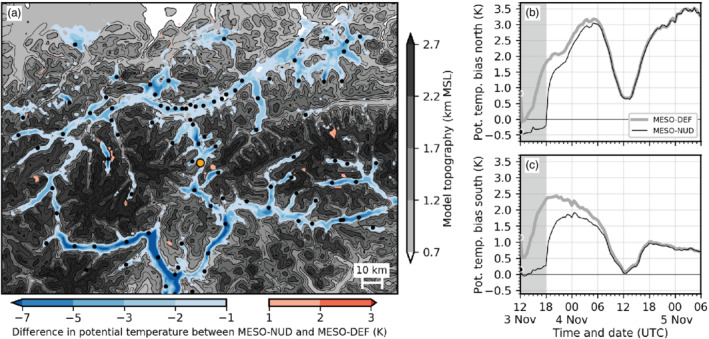
(a) Difference in potential temperature (colour shading, K) at the lowest model level (approximately 10 m agl) between MESO‐NUD and MESO‐DEF at 1800 UTC on 3 November (lead time +6 hr). Negative values (blue) correspond to lower potential temperature in MESO‐NUD than in MESO‐DEF. Values between –1 and +1 K are not shown. MESO model topography is shown as grey shading. Thick (thin) black contours indicate the topography with an interval of 500 (250) m amsl. Small black dots denote AWS locations which contribute data for observation nudging. The orange dot represents the location of Sattelberg at the main Alpine crest (SAT; Figure 1b). (b) and (c) illustrate time series of the mean error (bias in K) of the simulated air temperature at the lowest model level of MESO‐NUD and MESO‐DEF compared to all AWS observations used for nudging (b) north and (c) south of the main Alpine crest. Grey shading illustrates the time period of active observation nudging. The grey (black) marker at 1200 UTC on 3 November (lead time +0 hr) illustrates the mean bias related to the extrapolation of the input data (Appendix B).

The mesoscale WRF simulation is initialized at 1200 UTC on 3 November while the LES is initialized 6 hr later at 1800 UTC (Table 1). However, it was found that the default mesoscale simulation MESO‐DEF does not capture the CAP formation in the Inn Valley from 1200 to 1800 UTC but develops a warm bias of about 3.5 to 5 K compared to surface observations (Appendix A; Figure A1). Using this mesoscale WRF data would result in inaccurate initial conditions for the LES. Hence, a second mesoscale simulation (MESO‐NUD; Table 1) has been conducted which applies data assimilation by observation nudging (Liu *et al*., 2006; Liu *et al*., 2008). MESO‐NUD and MESO‐DEF share the same domain (Figure 1a) and model set‐up (Section 2.2). Observation nudging in MESO‐NUD is active only in the first 6 hr of the simulation until the initialization time of the LES and uses temperature and humidity observations from valley floor locations below 1,200 m amsl (Figure A1). More details on the observation nudging and its impact are given in Appendix A.

**TABLE 1:** Mesoscale simulations (MESO) and large‐eddy simulations (LES) conducted in this study

**Name**	**Simulation time**	**Initial and boundary conditions**	**Comments**
**MESO‐DEF**	1200 UTC 3 November – 2100 UTC 5 November 2017	6‐hourly ECMWF HRES analysis	Δx=1 km; deficiencies in CAP formation (Section 2.3)
**MESO‐NUD**	1200 UTC 3 November – 2100 UTC 5 November 2017	6‐hourly ECMWF HRES analysis plus surface observations in the target area	Δx = 1 km; observation nudging active until lead time +6 hr
**LES‐dx200**	1800 UTC 3 November– 0000 UTC 5 November 2017	30 min MESO‐NUD output (offline nesting via ndown)	Δx = 200 m; represents outer coarse‐resolution LES domain
**LES‐dx40**	1800 UTC 3 November – 0000 UTC 5 November 2017	Online, one‐way nesting in LES‐dx200	Δx = 40 m; represents inner high‐resolution LES domain

### 2.4 Model diagnostics

This section gives a brief overview of the new diagnostic variables introduced in the WRF model to analyze the foehn–CAP interactions. More details are given in Appendix B. The modified WRF model source code and the set‐up files of all simulations can be found in Umek (2020).

The LES is considered to resolve the largest scales of turbulence represented by large eddies. However, due to the spatial discretization in numerical models, the (partly) turbulent flow variables can be regarded as an average over a model grid box. Therefore, the grid‐box average for a flow variable $\tilde{a}$ is influenced by the horizontal and vertical mesh size of the numerical model (Wagner *et al*., 2014). Such a grid‐box average $\tilde{a}$ can be separated into a contribution by the mean flow *A* and a fluctuation $a^{\prime }$ due to the resolved part of turbulence $\tilde{a}\left(x,y,z,t\right)=A\left(x,y,z,t\right)+a^{\prime }\left(x,y,z,t\right)$. Time‐averaging of $\tilde{a}$ over the interval *T* is performed during model integration and yields

(1) $$A\left(x,y,z,t\right)=\bar{\tilde{a}}\left(x,y,z,t\right)=\int_{t-T}^{t}\tilde{a}\left(x,y,z,\hat{t}\right)d\hat{t},$$

with the overbar denoting the averaging operator. Due to the high spatial heterogeneity of the flow in real‐case complex terrain, the ensemble average in this study is approximated by a time averaging only (Equation 1), whereas in idealized LES studies often time and space averaging is used (e.g., Schmidli, 2013; Wagner *et al*., 2015; Leukauf *et al*., 2017). By using Equation 1, block averages for the three wind components and potential temperature (*U*, *V*, *W*, Θ, respectively) and other parameters (Appendix B) are calculated for *T* = 30 min during model integration based on data from each model time step.

Approximating the ensemble average with Equation 1 allows Reynolds‐averaging rules to be applied on the product of two variables $\tilde{a}$ and $\tilde{b}$ (Wyngaard, 2010). The explicitly resolved part (RES) of the covariance between these variables in the numerical simulation is then calculated by ${\bar{a^{\prime }b^{\prime }}}_{\text{RES}}=\bar{\tilde{a}\;\tilde{b}}-A\;B$. If one of the variables is a wind component, the covariance represents a resolved turbulent flux. Similarly, ${\bar{a^{\prime 2}}}_{\text{RES}}$ denotes the resolved variance of the quantity $\tilde{a}$. The explicitly resolved part of the TKE is then diagnosed by ${\text{TKE}}_{\text{RES}}=0.5\left({\bar{u^{\prime 2}}}_{\text{RES}}+{\bar{v^{\prime 2}}}_{\text{RES}}+{\bar{w^{\prime 2}}}_{\text{RES}}\right)$. The SGS part of the turbulence and turbulent fluxes are parametrized in the model (Section 2.2). Instantaneous SGS turbulent fluxes and velocity variances are diagnosed at every model time step following Deardorff (1980). Here, SGS contributions are also averaged for 30‐min intervals to be consistent with the resolved part. Hence, the total (TOT) turbulent quantity in the LES is then given by the sum of the two components RES and SGS, e.g., for the total vertical heat flux ${\bar{w^{\prime }\theta^{\prime }}}_{\text{TOT}}={\bar{w^{\prime }\theta^{\prime }}}_{\text{RES}}+{\bar{w^{\prime }\theta^{\prime }}}_{\text{SGS}}$.

To analyse CAP formation and erosion, individual contributions to the total rate of change of the potential temperature (i.e., tendencies in K·s^−1^) at individual grid points are diagnosed during model integration. While the thermodynamic equation is formulated in flux form in the WRF model together with a proprietary *moist* potential temperature (Skamarock *et al*., 2019), our newly added model diagnostics use the advective form and the standard definition of potential temperature. Applying the usual Boussinesq approximation to the flux form of the thermodynamic equation and the time‐averaging as defined in Equation 1 yields

(2) $$\bar{\frac{\partial \tilde{\theta }}{\partial t}}=-\bar{\tilde{v}\cdot \nabla \tilde{\theta }}+\bar{S_{\text{SGSTRB}}}+\bar{S_{\text{RAD}}}+\bar{S_{\text{MP}}},$$

with $\tilde{v}$ denoting the three‐dimensional wind velocity. The last three terms on the right‐hand side denote time‐averaged potential temperature tendencies resulting from SGS turbulence (SGSTRB), radiation (RAD), and microphysical processes (MP). As mentioned above, SGS turbulence is treated fully three‐dimensionally in the LES and diagnostic terms are split in all three Cartesian directions (Appendix B). Moreover, the approximation of the ensemble average by Equation 1 allows the time‐average of the total diagnosed advection of potential temperature $-\bar{\tilde{v}\cdot \nabla \tilde{\theta }}$ in Equation 2 to be split into a mean flow advection (MADV) and a resolved turbulent advection (RESTRB)

(3) $$\begin{array}{cc} -\bar{\tilde{v}\cdot \nabla \tilde{\theta }} & =\underset{\text{MADV}}{\underbrace{-U\frac{\partial \Theta }{\partial x}-V\frac{\partial \Theta }{\partial y}-W\frac{\partial \Theta }{\partial z}}}\\ & \;\underset{\text{RESTRB}}{\underbrace{-\bar{u^{\prime }\frac{\partial \theta^{\prime }}{\partial x}}-\bar{v^{\prime }\frac{\partial \theta^{\prime }}{\partial y}}-\bar{w^{\prime }\frac{\partial \theta^{\prime }}{\partial z}}}}. \end{array}$$

During model integration, the time‐averaged total advection of potential temperature (left‐hand side of Equation 3) and MADV of Equation (3) are explicitly diagnosed. The contribution of RESTRB is then calculated during post‐processing as a residual for each Cartesian component.

## 3 SYNOPTIC AND MESOSCALE OVERVIEW

### 3.1 Synoptic situation during PIANO IOP 2

**FIGURE 2:**
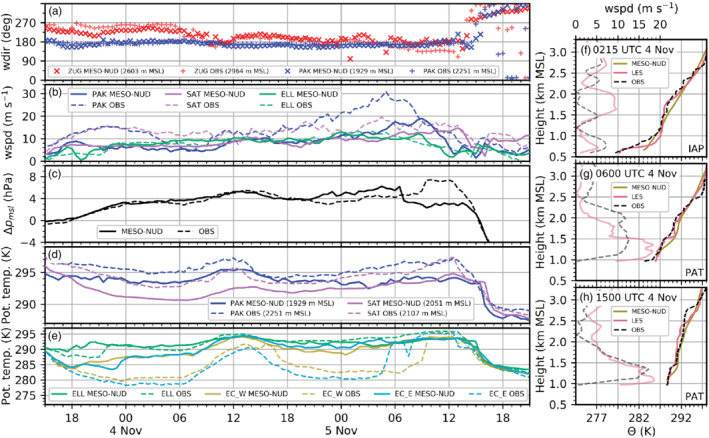
Observations (dashed) and WRF model data (solid) between 1500 UTC on 3 November and 2100 UTC on 5 November 2017. (a)–(e) show time series at surface sites and (f)–(h) vertical profiles; model data in (a)–(e) are from MESO‐NUD and taken from the lowest model level at 10 m agl. (a) Wind direction at ZUG and PAK. (b) Wind speed at PAK, SAT and ELL. (c) Difference of the air pressure reduced to mean sea level between Bozen (BOZ) and Innsbruck Airport (IAP) $\Delta p_{\text{msl}}=p_{\text{msl}}$(BOZ)‐*p*_msl_(IAP). Potential temperature at (d) PAK and SAT and (e) in the Wipp Valley (ELL) and the Inn Valley (EC_W, EC_E). Vertical profiles of potential temperature (dark colours) and wind speed (light colours) are based on model data (solid) and radiosonde ascents (dashed) launched on 4 November at (f) 0215 UTC at IAP and at (g) 0600 UTC and (h) 1500 UTC at PAT. Data from MESO‐NUD and LES‐dx40 are interpolated in time and space along the radiosonde trajectory. Wind speed in (f)–(h) is only shown for LES‐dx40. Figure 1b,c give locations

This section provides a brief overview of the synoptic development of IOP 2 from observations. A more detailed analysis is provided in Haid *et al*. (2020). On 3 November 2017, the Alpine region was located at the leading edge of a large‐scale trough (not shown). Differential air‐mass advection across the main Alpine ridge led to warmer air at low levels north of the Alps compared to the Alpine southside. This temperature difference induced a cross‐Alpine pressure gradient with higher pressure south of the main Alpine crest. Haid *et al*. (2020) showed that south foehn was present at the mountain peak station Patscherkofel (PAK; 2,251 m amsl; Figure 1b) after 1100 UTC on 3 November, while at the higher Zugspitze (ZUG; 2,964 m amsl; Figure 1b) westerly winds prevailed (e.g., after 1500 UTC 3 November in Figure 2a). This restriction of cross‐Alpine flow to heights below the main crest level classifies the initial phase of IOP 2 as *shallow foehn*. Winds at the Inn and Wipp Valley floor were weak around noon on 3 November and hence did not represent foehn (not shown). After 2100 UTC on 3 November, south foehn developed at Ellboegen (ELL; 1,080 m amsl), a station in the northern part of the Wipp Valley (Figure 1c). There, the foehn onset was indicated by an increase in observed wind speed and potential temperature (Figure 2b,e).

From 3 to 5 November the large‐scale foehn forcing gradually intensified, as illustrated by an increase in the observed cross‐Alpine pressure gradient (Figure 2c). Around 0600 UTC on 4 November, winds at ZUG veered to southwest, resulting in a transition to *deep foehn* (Figure 2a). This led to an increase in the foehn layer depth, a stronger foehn jet and a reduced static stability of the foehn layer in the Wipp Valley (Figure 2g,h; Haid *et al*., 2020). Observations indicate a partial foehn breakthrough in the western part of Innsbruck between 1500 and 1700 UTC on 4 November (EC_W in Figure 2e; Haid *et al*., 2020) and a re‐establishment of the CAP during the night from 4 to 5 November (EC_W and EC_E in Figure 2e). An intermittent foehn breakthrough to the Inn Valley had already happened at about 0500 UTC 5 November around EC_E and the full foehn breakthrough at the Inn Valley floor was observed before noon on 5 November, the second day of IOP 2. Observed potential temperature at EC_W and EC_E strongly increased and finally matched the potential temperature at ELL, which illustrates well‐mixed conditions in the lower part of the Inn Valley atmosphere (Figure 2e). More details on the observed CAP heterogeneity on the second day of IOP 2 can be found in Haid *et al*. (2020). The foehn event was finally terminated by the arrival of a cold front in the Inn Valley at about 1400 UTC on 5 November (e.g., Figure 2e).

### 3.2 Mesoscale WRF simulation MESO‐NUD

After 3 hr of model spin‐up (not shown), the MESO‐NUD simulation captures the large‐scale flow pattern reasonably well and reproduces the shallow foehn on 3 November 2017. For example, MESO‐NUD reproduces the southerly flow at PAK during foehn and the wind shift associated with the cold front passage in the afternoon of 5 November (Figure 2a). Additionally, the transition from shallow to deep foehn with a wind shift at ZUG and the evolution and magnitude of the large‐scale pressure gradient between BOZ and IAP on 3 and 4 November are captured well (Figure 2a,c). Small differences in wind directions at ZUG are present which are considered to be caused by differences in the local flow pattern due to differences between the smooth model terrain and true complex topography at the Zugspitze observatory (Figure 2a). Wind speeds are mostly underestimated at SAT and PAK on the lowest model level at 10 m agl (Figure 2b). However, increasing wind speed between 0600 and 1200 UTC on 4 November at PAK during the change from shallow to deep foehn is captured by MESO‐NUD.

Potential temperatures at PAK and SAT exhibit a systematic cold bias throughout the simulation of up to 3 K (Figure 2d). However, the height difference between the model topography and the observations is around 300 m at PAK and 50 m at SAT which partly contributes to this cold bias, especially at PAK. The reason for the generally higher observed and simulated potential temperature at PAK compared to SAT is most likely twofold. On the one hand, PAK is affected by subsidence warming on the leeward side of the main Alpine crest (Figure 1b). On the other hand, the slightly higher potential temperature difference between PAK and SAT in the simulation compared to observations suggest that the foehn flow is too stably stratified in MESO‐NUD which further enhances subsidence warming (Figure 2d). Comparing potential temperature measured by a radiosonde launched at PAT with MESO‐NUD data interpolated onto the radiosonde trajectory confirms a too stable stratification of the foehn flow (Figure 2f,g). Furthermore, MESO‐NUD exhibits a too early onset of foehn at the Wipp Valley floor, as illustrated by the earlier increase in potential temperature after 1800 UTC on 3 November at ELL in the simulation (Figure 2e). After the observed onset of foehn at ELL (2200 UTC on 3 November), wind speeds and potential temperature of MESO‐NUD are in reasonable agreement with observations (Figure 2b,e).

During the phase of observation nudging until 1800 UTC on 3 November (Section 2.3 and Appendix A), MESO‐NUD closely reproduces the temperature at the Inn Valley floor (Figure 2e). However, simulated potential temperatures later start to increase at the Inn Valley floor whereas cooling continued in the observations for more than 6 hr (Figure 2e). Therefore, MESO‐NUD is neither able to intensify nor to maintain the CAP. The vertical profile of potential temperature measured by a radiosonde launched at 0215 UTC on 4 November at IAP illustrates a pronounced CAP below 1,200 m amsl characterized by enhanced stability (Figure 2f). In contrast, the profile of MESO‐NUD exhibits a less stable low‐level stratification in the Inn Valley with a nearly constant stability up to crest height, which results in a near‐surface temperature bias of about 6 to 8 K at the Inn Valley floor around 0215 UTC on 4 November (Figure 2e,f). Simulated foehn onset in the Inn Valley takes place during the night from 3 to 4 November in MESO‐NUD (Figure 2e). The warm bias of MESO‐NUD compared to observations at the Inn Valley floor increases to 7–10 K at 0600 UTC on 4 November. Furthermore, no CAP forms in the Inn Valley in MESO‐NUD during the night from 4 to 5 November and, hence, foehn prevails and leads to a strong warm bias compared to observations at EC_W and EC_E (Figure 2e). Simulated potential temperatures in MESO‐NUD agree reasonably well with observations in the Inn Valley after the observed foehn penetration after 0600 UTC on 5 November (Figure 2e).

Although MESO‐NUD captures the mesoscale cross‐Alpine pressure difference between BOZ and IAP reasonably well, there is a prominent negative bias of about −4 hPa between 0700 and 1400 UTC on 5 November (Figure 2c). While the observations show a sudden *increase* in this pressure difference due to the removal of the CAP in the Inn Valley (Haid *et al*., 2020), the model exhibits a sudden *decrease*. Inspection of the stratification above the Inn Valley reveals that the latter is caused by a change in the simulated gravity‐wave field near IAP (not shown). As the wave crest shifts upstream and establishes above the valley centre, the surface pressure at the valley floor increases and, therefore, the pressure difference between BOZ and IAP decreases. Hence, the discrepancy in the simulated and observed pressure gradient is most likely caused by a discrepancy in the local gravity‐wave response.

In summary, MESO‐NUD is able to reproduce the mesoscale forcing of the foehn event, especially on 3 and 4 November (Figure 2a–d), but fails in enhancing and maintaining the CAP in the Inn Valley which results in a too early foehn breakthrough (Figure 2e,f). The poor performance of the mesoscale WRF simulations is in contrast to simulations of Zängl *et al*. (2004b) with a horizontal grid spacing of Δx = 800 m. However, the performance is comparable to operational forecast models, such as COSMO‐1 of MeteoSwiss which also does not capture the strength of the CAP correctly (Sandner, 2020). This calls for an LES to better represent and investigate the foehn–CAP interaction in the Inn Valley. As shown in Section 2.3 and Appendix A, observation nudging leads to more realistic near‐surface temperatures in the Inn Valley at 1800 UTC on 3 November which represents the start time of the LES. Hence, initial conditions of the LES provided by MESO‐NUD are not affected by a systematic temperature bias. The performance of the MESO‐NUD simulation in the Inn Valley degrades after the simulated foehn penetration during the night from 3 to 4 November and is poor until the passage of the cold front on the afternoon of 5 November. However, since the two LES domains cover most parts of the Inn Valley, the quality of CAP maintenance and intensification in the LES is not strongly dependent on the performance of MESO‐NUD.

## 4 REGIONAL CHARACTERISTICS OF THE FOEHN IN THE LES

### 4.1 Foehn in the north–south aligned Wipp Valley

**FIGURE 3:**
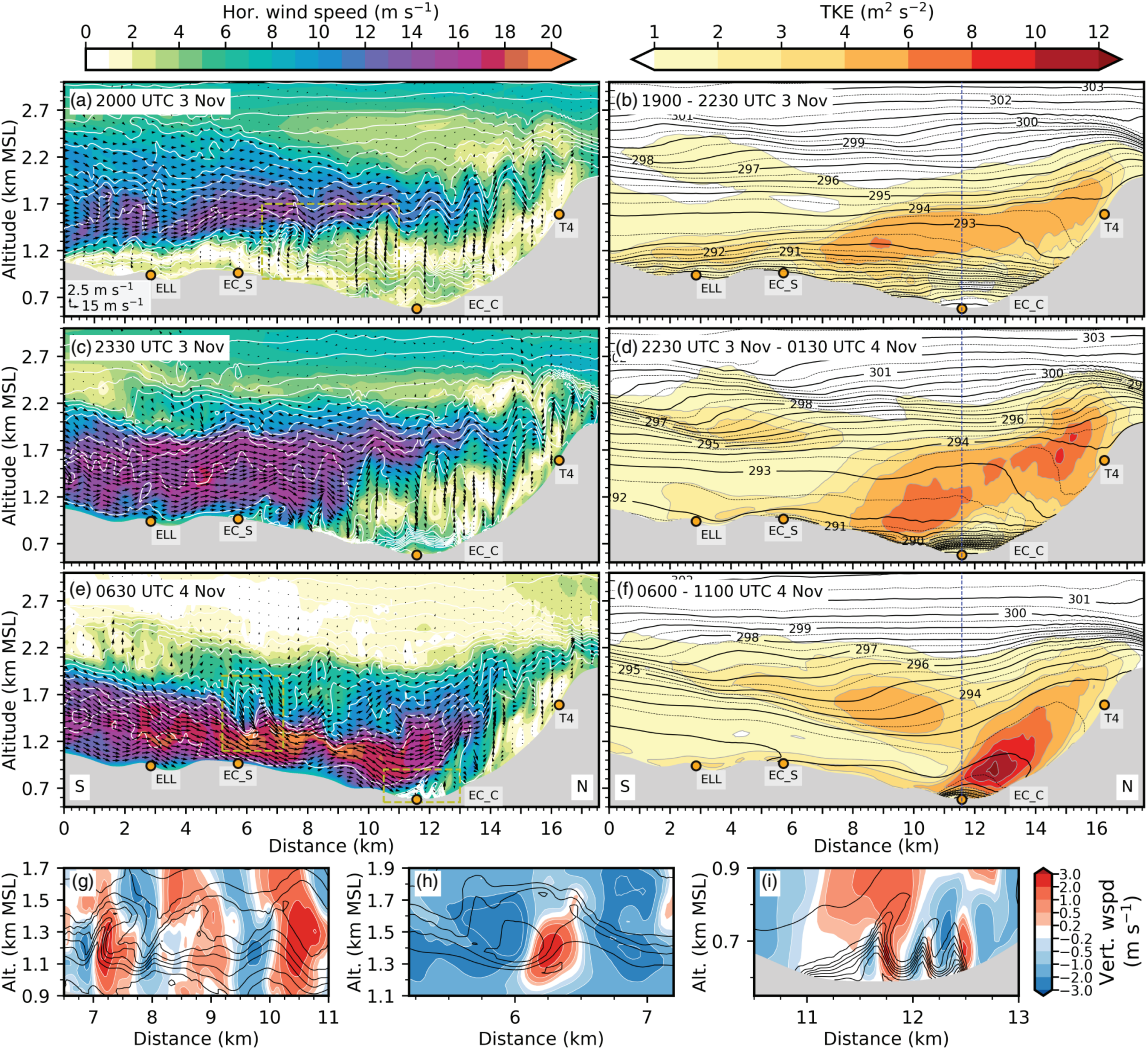
Vertical cross‐section of LES‐dx40 data from south to north along the transect WIPP (Figure 1c). Grey shading indicates the model topography. Annotated orange dots show points of interest as in Figure 1c. Instantaneous potential temperature (white lines; 1 K increments), absolute horizontal wind speed (coloured contours; 1 m·s^−1^ increments) and transect‐parallel wind vectors are shown for (a) 2000 UTC on 3 November, (c) 2330 UTC 3 November, and (e) 0630 UTC on 4 November. A reference vector is given in (a). (b), (d) and (f) illustrate the mean total TKE (coloured contours) and the mean potential temperature (black solid and dotted lines; 0.5 K increments) averaged for the periods (b) 1900–2230 UTC on 3 November, (d) 2230 UTC on 3 November to 0130 UTC on 4 November, and (f) 0600–1100 UTC on 4 November. The dashed vertical line in (b), (d), and (e) indicates the intersection with the transect INN (Figure 1c). (g)–(i) illustrate Kelvin–Helmholtz instability based on the instantaneous vertical wind speed (coloured contours) and potential temperature (black lines; 1 K increments) for three subdomains at (g) 2000 UTC on 3 November and (h)–(i) 0630 UTC on 4 November. The location of subdomain (g) is indicated by a yellow dashed rectangle in (a). The locations of subdomains (h) in the Wipp Valley and (i) in the Inn Valley are indicated in (e)

**FIGURE 4:**
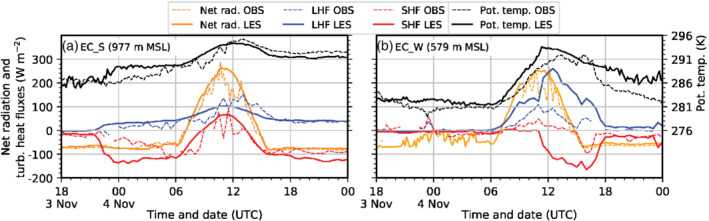
Time series of observed (dashed lines) and simulated (solid lines, data from LES‐dx40) net radiation (orange) and turbulent sensible (SHF, red) and latent heat (LHF, blue) fluxes at the surface, as well as potential temperature (black) for (a) EC_S and (b) EC_W. Positive heat fluxes are directed upwards, away from the surface. Net radiation and potential temperature are shown as 10‐min averaged values and turbulent fluxes are based on 30‐min block averages. Gaps in the observations are present where data have been removed by quality control

The following analysis is based on the LES‐dx40 run initialized at 1800 UTC on 3 November and restricted to the first day of IOP 2 until 0000 UTC on 5 November. Afterwards, the deficiencies of the LES become larger, despite the overall improvement of the foehn evolution compared to MESO‐NUD. Figure 3 shows LES‐dx40 data along the transect WIPP at the Wipp Valley exit and across the Inn Valley (Figure 1). By 2000 UTC on 3 November, an elevated foehn jet has formed in the north–south aligned Wipp Valley, while weak winds prevail below 1.2 to 1.5 km amsl in the Wipp and Inn Valleys (Figure 3a). A CAP is present in the Inn Valley and reaches southward into the Wipp Valley beyond EC_S and ELL (mean isentropes in Figure 3b). Until about 2230 UTC on 3 November, the foehn flow also becomes established in the Wipp Valley near the surface, except for the last 2 km near the valley exit (Figure 3c and Figure 4a) where the CAP persists (Figure 3c,d). At PAK, the LES‐dx40 exhibits a cold bias of 1 to 2.5 K before noon on 4 November which reduces to 0.5 K afterwards (not shown). However, one part of this cold bias is attributable to the height difference between the peak of PAK in LES‐dx40 (2,130 m amsl) and the AWS location (2,251 m amsl). Wind speed at PAK is underestimated in LES‐dx40, similar to MESO‐NUD as mentioned in Section 3.2. Potential temperature at SAT (representing the flow over the main Alpine crest) exhibits a cold bias of 1.5 to 3 K before noon on 4 November and 1 K afterwards (not shown). The difference between the model topography and the observation height is also around 100 m at SAT.

**FIGURE 5:**
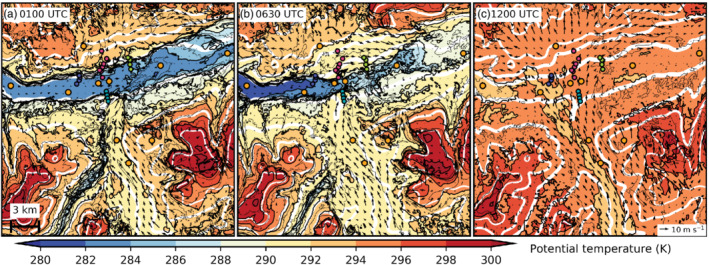
Instantaneous potential temperature (colour contours, 2 K interval) of LES‐dx40 on the lowest model level at (a) 0100 UTC, (b) 0630 UTC, and (c) 1200 UTC on 4 November. Black contours denote isentropes with a 1 K interval. Horizontal wind on the lowest model level is depicted by arrows with a reference vector in (c). Topography of the LES is indicated by white contour lines for 700, 1,200, 1,700, 2,200 m amsl (cf. thick black contour lines in Figure 1c). The thickest (thinnest) white contour line represents 700 (2200) m amsl. Orange dots identify the location of observation sites and small coloured dots denote slope‐profile stations as shown in Figure 1c. A length‐scale is given in (a)

Within the Wipp Valley, the foehn flow exhibits a high spatial variability. Foehn breakthrough at ELL is observed by a rapid temperature increase at 2200 UTC on 3 November (Figure 2e) and nearly perfectly reproduced by the diagnosed 2 m temperature of LES‐dx40 (not shown). However, only 5 km downstream of ELL at EC_S, the observed onset of foehn is a gradual process with slowly rising temperature (Figure 4a). Consequently, in the observations the point in time of reaching the foehn temperature is delayed by about 7 hr until 0500 UTC on 4 November. Haid *et al*. (2020) hypothesize that the slower increase in temperature at EC_S is related to cold‐air outflow from the Stubai Valley, a tributary west of ELL (Figure 1c) as supported by a westerly wind component observed during this pre‐foehn stage. In LES‐dx40 such an outflow does indeed occur (e.g., at 0100 UTC on 4 November in Figure 5a) but not at the grid point closest to EC_S. There, the LES indicates an abrupt temperature increase due to the onset of foehn (Figure 4a) at about the same time as at ELL. Therefore, the cold‐air outflow and its effect is presumably underestimated by the model. After 0500 UTC on 4 November, simulated and observed potential temperature of the foehn at EC_S are in good agreement (Figure 4a). With the gradual onset of foehn at EC_S, the observed negative surface sensible heat flux intensifies from −20 to −100 W·m^−2^ at 0500 UTC on 4 November (Figure 4a). In line with the earlier and more sudden foehn breakthrough in LES‐dx40, the simulated surface sensible heat flux rapidly reaches −100 to −140 W·m^−2^. These values are comparable to previously observed and simulated surface sensible heat fluxes during foehn in the Wipp Valley (Zängl *et al*., 2008). Observed and simulated surface latent heat fluxes and net radiation are in reasonable agreement at EC_S (Figure 4a).

With increasing mesoscale foehn forcing during the night from 3 to 4 November (cf. Figure 2c), the foehn jet in the Wipp Valley intensifies (cf. Figure 3c,e). After 2230 UTC on 3 November, the CAP in LES‐dx40 disappears in the northern Wipp Valley but still prevails with enhanced mean static stability in the Inn Valley (cf. Figure 3b,d). A comparison of the simulated and observed vertical potential temperature structure in the Inn valley near IAP at 0215 UTC on 4 November is shown in Figure 2f. The increase in potential temperature over the depth of the CAP in LES‐dx40 is comparable to the observations but the simulated CAP is about 200 to 300 m shallower. The observed near‐neutral stratification of the foehn flow above the CAP is captured by LES‐dx40 (Figure 2f) but not by MESO‐NUD (Section 3.2). Simulated and observed wind speed is rather weak in the Inn Valley with pre‐foehn westerlies below about 1 km amsl (Figure 2f).

Around 0600 UTC on 4 November, the transition from shallow to deep foehn occurs (cf. Figure 2a). The simulated foehn jet in the Wipp Valley is characterized by an approximately 1 km deep layer topped by a temperature inversion (Figure 3e). The latter becomes deeper but weaker northward towards the Inn Valley due to turbulent mixing resulting from strong shear at the top of the foehn jet (Figure 3e,f). Figure 2g shows a comparison of vertical profiles of potential temperature and wind speed measured by a radiosonde launched at PAT near EC_S at 0600 UTC on 4 November and the corresponding LES‐dx40 data. Overall, the LES captures the stratification of the foehn jet reasonably well, apart from a very shallow and strongly stable layer near the surface. Discrepancies at higher levels are partly caused by the highly transient flow field in the Wipp Valley. For example, the deformation of the capping inversion due to shear flow instability leads to a time dependent temperature field (Figure 3e,h). This transient behaviour may also affect instantaneous wind speeds. For example, the model sounding exhibits a pronounced low‐level jet maximum of about 18 m·s^−1^ whereas the observed sounding shows a deeper and weaker jet with winds up to 12 m·s^−1^ (Figure 2g).

During the phase of fully developed deep foehn in the afternoon of 4 November, the observed potential temperature sounding in the Wipp Valley indicates a four‐layer structure with two elevated inversions (Figure 2h). A 400 m deep and nearly mixed layer above the surface is capped by a shallow, more stably stratified inversion at around 1.4 km amsl. Above, the valley atmosphere is again weakly stratified up to about 2.8 km amsl where the second shallow inversion is located (Figure 2h).

LES‐dx40 reproduces this vertical structure of the sounding at 1500 UTC on 4 November reasonably well but places the upper inversion around 300 to 400 m too low (Figure 2h). A pronounced low‐level wind speed maximum exceeding 15 m·s^−1^ is observed and reproduced by LES‐dx40 (Figure 2h). The weakly stratified layer above is characterized by strong vertical wind shear and associated strong turbulent mixing between the two inversions. In the upper Wipp Valley, the foehn jet is topped only by a single inversion (e.g., southward of ELL in Figure 3c) and the formation and breaking of K‐H waves (e.g., Figure 2h) leads to the splitting of this inversion with the formation of a weakly stratified layer mentioned above in between (e.g., Figure 3e). The lower inversion limits the strongest part of the foehn jet which eventually reaches the Inn Valley floor (cf. Figure 3e). The upper inversion bounds the upper part of the foehn which flows over the Nordkette (e.g., Figure 3e,f).

### 4.2 Foehn in the west–east aligned Inn Valley

As mentioned in Section 4.1, a CAP is present in the west–east orientated Inn Valley while south foehn developed in the north–south aligned Wipp Valley in the night from 3 to 4 November. This section provides an overview of the evolution of the foehn flow and its breakthrough in the Inn Valley simulated in LES‐dx40.

From 2230 UTC on 3 November to 0130 UTC on 4 November, simulated potential temperatures in the CAP at about 10 m above the Inn Valley floor are quite homogeneous (e.g., Figure 5a). However, LES‐dx40 exhibits a weak warm bias of 2 to 4 K at the Inn Valley bottom which reduces towards the end of the night (Figure 4b). Foehn air at the Wipp Valley floor has a potential temperature of about 290 to 291 K and is therefore up to 9 K warmer than at the Inn Valley floor. In the model, a shallow layer of low stratus forms above 50 m agl west of SL74 (not shown), but such cloud formation was not observed. Instead, dew formed on the radiation sensors of EC_W and EC_E which led to erroneous radiation recordings (cf. missing observed net radiation until about 1000 UTC on 4 November in Figure 4b). Simulated net radiation is affected by the spurious cloud formation between 2100 UTC on 3 November and 0430 UTC on 4 November and cooling is reduced west of the city compared to observations especially before 0000 UTC on 4 November (Figure 4b). Observed and simulated half‐hourly averaged sensible heat fluxes at EC_W are rather small (−10 to −20 W·m^−2^) and in good agreement during the night from 3 to 4 November (Figure 4b). A positive surface sensible heat flux (up to 40 W·m^−2^) is present at urban land‐use areas in LES‐dx40 during night‐time (not shown).

**FIGURE 6:**
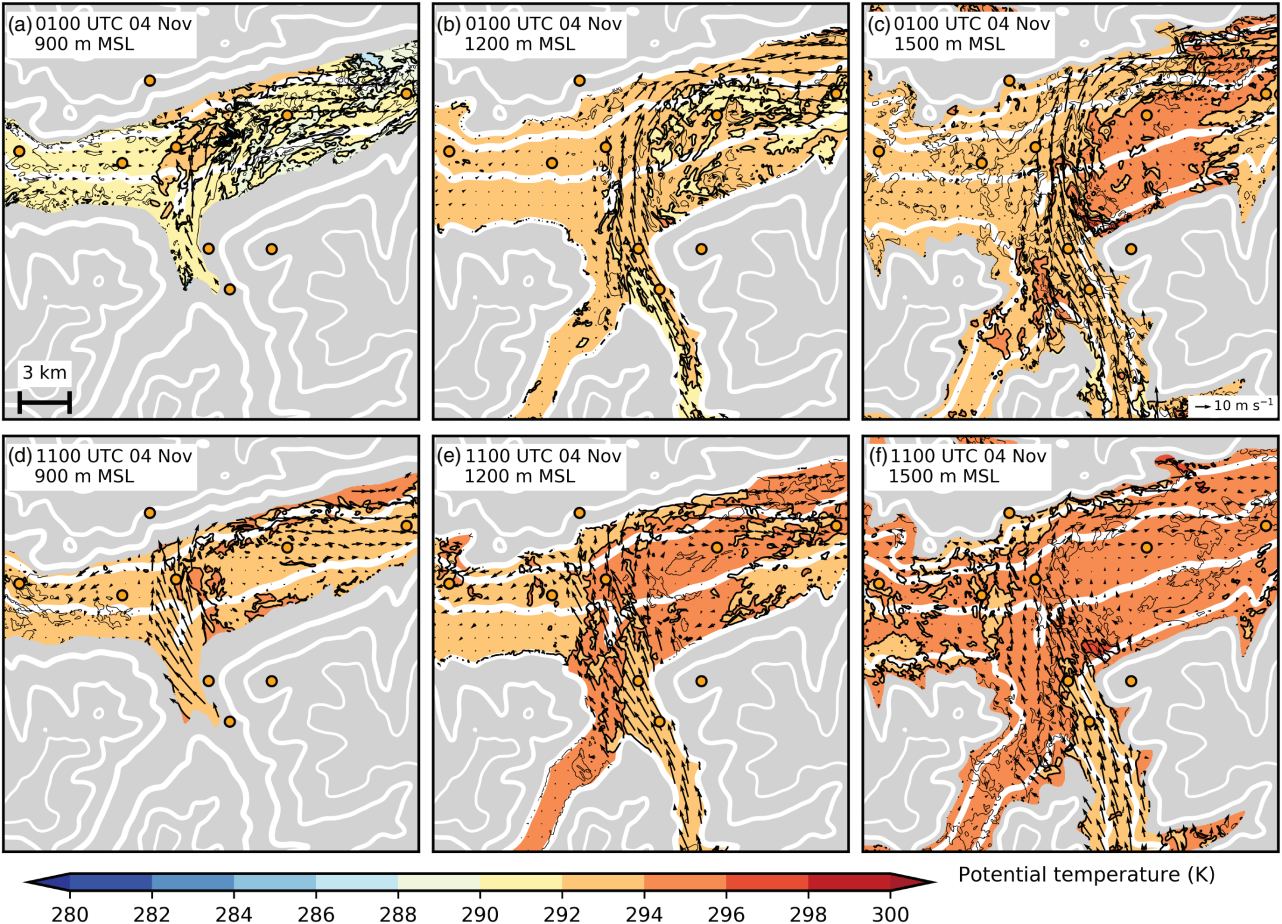
As Figure 5 but interpolated to constant height levels at (a, d) 900 m amsl, (b, e) 1,200 m amsl, and (c, f) 1,500 m amsl for (a–c) 0100 UTC and (d–f) 1100 UTC on 4 November. Grey shading indicates the model topography above the respective height level

**FIGURE 7:**
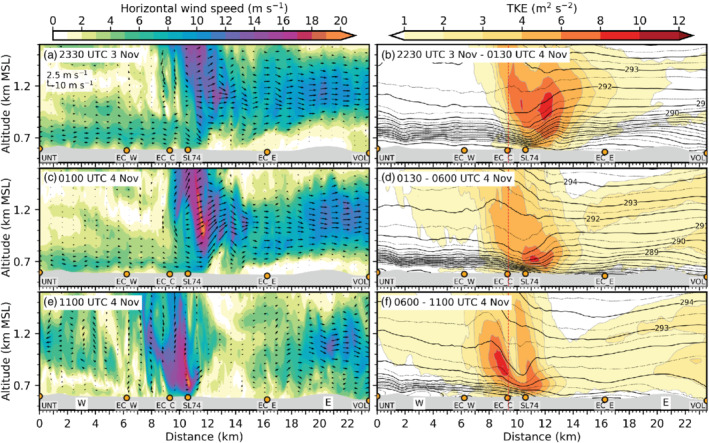
As Figure 3a–f but from west to east along the transect INN (Figure 1c) and at (a) 2330 UTC on 3 November, (c) 0100 UTC and (e) 1100 UTC on 4 November. (b), (d) and (f) illustrate the mean total (resolved and SGS) TKE and mean isentropes averaged for the periods 2230–0130 UTC, 0130–0600 UTC and 0600–1100 UTC on 4 November, respectively. The dashed vertical line in (b), (d), and (f) indicates the intersection with the transect WIPP in Figure 3

The foehn jet emanating from the Wipp Valley crosses the Inn Valley above the CAP, illustrated by strong winds above 1 km amsl in the along‐valley cross‐section of Figures 7a and 3c. Subsequently, the foehn jet impinges on the Nordkette range north of Innsbruck (Figure 1c). There, flow deflection results in a pronounced foehn branch towards the lower (eastern) Inn Valley after about 2230 UTC on 3 November (Figure 6a–c). A secondary elevated wind maximum east of the city between EC_E and VOL (Figure 7a,c,e) is related to this eastward deflected foehn which re‐enters the cross‐section from the north. During the night and with the change from shallow to deep foehn after 0600 UTC on 4 November, simulated foehn wind speed in the Wipp Valley increases (cf. Figure 3c,e). Subsequently, flow deflection at the Nordkette also intensifies, where potential temperatures near SP_N and SP_NE match the temperature of the foehn in the Wipp Valley (Figure 5b). Moreover, flow deflection towards the upper Inn Valley (westward) increases after the change to deep foehn (cf. Figure 6a,b,d,e). However, with the change to deep foehn, the depth of the foehn jet increases and the inversion capping the foehn jet ascents to around 2.4 km amsl in LES‐dx40 (cf. Figure 2g,h) which is several hundred metres above crest height of the Nordkette north of Innsbruck in the LES. Therefore, more foehn air is hypothesized to flow over the Nordkette and flow deflection is slightly decreased at higher levels, e.g., at 1,500 m amsl (cf. Figure 6c,f).

Mean total (resolved and SGS) TKE exceeds 8 m^2^·s^−2^ in the transition zone between the CAP and the foehn jet over the city centre (Figure 7b,d,f). During the first half of the night, the maximum mean total TKE is located more towards the southern portion of the Inn Valley and moves towards the Nordkette later in the night when foehn wind speed increases and the CAP becomes shallower (cf. Figure 3d,f). Moreover, mean total TKE increases east of the city during the second half of the night, especially close to the Inn Valley floor (cf. Figure 7b,d). Figure 5a,b illustrates that the Inn Valley CAP continues to cool west of Innsbruck between 0100 and 0630 UTC on 4 November (also Figure 4b), while it warms due to erosion east of Innsbruck. A stronger heterogeneity of the temperature and wind field in the east together with a stronger eastward foehn deflection illustrates the more vigorous foehn–CAP interaction and faster CAP erosion east than west of the city centre. By 1100 UTC on 4 November, the CAP in the Inn Valley is completely eroded (cf. Figure 5c) and foehn breakthrough first occurs near the city centre depicted by strong low‐level winds between EC_C and SL74 in Figure 7e.

With the simulated foehn breakthrough at EC_W (highest simulated potential temperature in Figure 4b), surface sensible heat fluxes turn strongly negative in LES‐dx40 (Figure 4b). Simulated potential temperature generally increases towards the time of the foehn penetration and is more homogeneous throughout the Inn and Wipp Valley afterwards, not only horizontally but also vertically (compare EC_C and PAK in Figure 5c). This is in contrast to the observations, which do not show a complete CAP erosion and foehn breakthrough at the Inn Valley floor on 4 November. As mentioned in Section 3.1, observations only document a localized foehn breakthrough in the area between SP_NW and SP_N, as well as at EC_W between 1500 and 1700 UTC on 4 November (observed potential temperature increase in Figure 4b). In contrast to the southerly winds (i.e., direct foehn) in LES‐dx40 (Figure 5c), variable wind directions are observed at the Inn Valley floor during the partial breakthrough on 4 November (Haid *et al*., 2020). An observed vertical sounding near Innsbruck Airport at 1500 UTC on 4 November exhibits a near neutral stratification in the lowest 1 km agl (not shown). Weak northerly winds prevail below 200 m agl (presumably deflected flow) and stronger southeasterly winds above (presumably the direct foehn jet) in the observations. Due to the direct foehn penetration in LES‐dx40, wind speeds near the surface are increased compared to observations, which is in line with much stronger turbulent heat fluxes at EC_W than the observed fluxes during the transient foehn period from 1500 to 1700 UTC on 4 November (Figure 4b).

After 1300 UTC on 4 November, observed and simulated potential temperature of the foehn air in the Wipp Valley start to decrease (Figure 4a). Cooling stops when net radiation reaches a minimum around 1530 UTC on 4 November. Observed and simulated potential temperature at EC_S remains almost constant thereafter with ongoing foehn in the Wipp Valley (Figure 4a), while observed potential temperature at the Inn Valley floor indicates the reformation of a CAP after 1700 UTC on 4 November (Figures 2e and 4b). In LES‐dx40, foehn air only intermittently reaches EC_W after 1800 UTC on 4 November, illustrated by large potential temperate fluctuations and a weakening of the simulated turbulent fluxes in Figure 4b. Further to the west (towards UNT in Figure 1c), CAP reformation is slightly stronger in LES‐dx40 but essentially no CAP forms near the city centre and EC_E (not shown). Therefore, LES‐dx40 is not able to correctly reproduce the foehn interruption during the night from 4 to 5 November. The analysis of LES data in the remainder of this work is restricted to the first day of IOP 2 before the foehn interruption and is focused on the CAP erosion.

### 4.3 Pre‐foehn westerlies in the Inn Valley

In the early night of 3 November simulated winds in the Inn Valley are weak (EC_C in Figure 3a). Later in the night, when the foehn flow in the Wipp Valley strengthens, winds in the stably stratified Inn Valley intensify also (Figure 3c). Enhanced down‐valley winds within the CAP are so‐called pre‐foehn westerlies. They are also illustrated in the vertical cross‐section along the Inn Valley in Figure 7a,c. The distinct near‐surface layer of westerly flow with a wind maximum at about 100 to 150 m agl is confined to the region from the city centre westward (cf. Figures 5a and 7a,c).

Around midnight, simulated potential temperature near the surface is nearly constant at the Inn Valley bottom between UNT and EC_E (e.g., Figure 5a). However, mean isentropes averaged from 2230 UTC on 3 November to 0130 UTC on 4 November in Figure 7b indicate higher static stability below 0.9 km amsl to the west than to the east of Innsbruck (cf. EC_W and EC_E). Furthermore, mean isentropes are slightly descending eastward from UNT towards EC_C, with the lowest height near the city centre co‐located with the wind maximum of the low‐level pre‐foehn westerlies (Figure 7a–d).

During the night from 3 to 4 November, observed pre‐foehn westerlies have maxima of about 7 m·s^−1^ at IAP (Figure 2f) and about 6–10 m·s^−1^ in the city centre (Figure 5c of Haid *et al*., 2020). In contrast, simulated pre‐foehn westerlies reach only about 5 m·s^−1^ (Figure 2f) and are continuously weakening during night‐time west of Innsbruck while at the same time intensifying near the city centre (cf. Figure 7a,c,e). During the whole night the wind maximum is located at the shallowest section of the CAP underneath the foehn jet exiting from the Wipp Valley (cf. Figure 7a–f). Until the simulated foehn breakthrough at 1100 UTC on 4 November, pre‐foehn westerlies in the west near EC_W have essentially ceased and are replaced by direct foehn from the southeast near the valley floor (cf. Figure 5c) and westward deflected foehn above (cf. Figures 6d–f and 7e).

### 4.4 Spatial heterogeneity of the Inn Valley CAP prior to the foehn breakthrough

**FIGURE 8:**
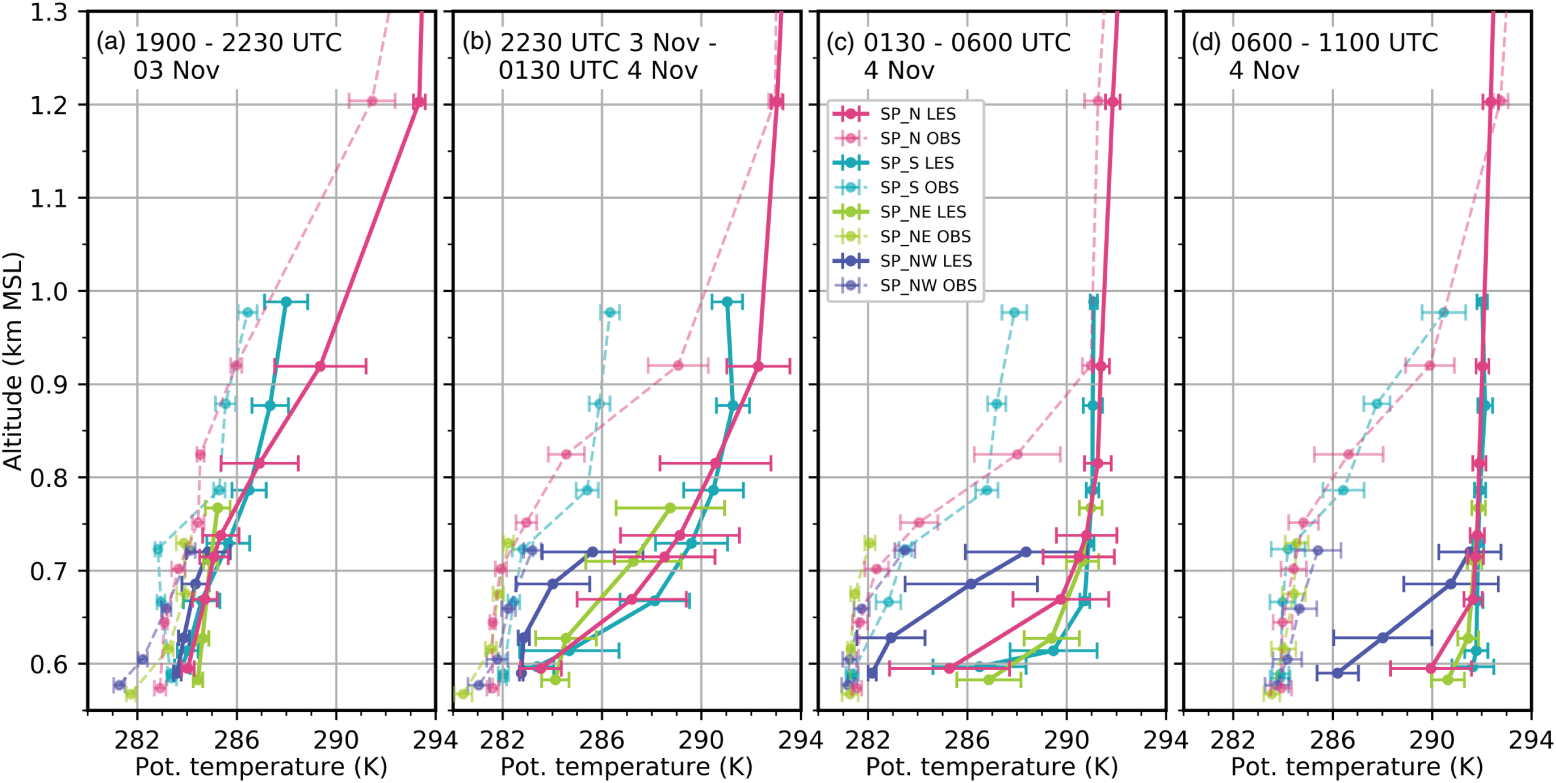
Pseudo‐vertical profiles of observed (light dashed) and simulated (LES‐dx40, dark solid) potential temperature along four different slopes averaged for the periods (a) 1900–2230 UTC on 3 November, (b) 2230 UTC–0130 UTC, (c) 0130–0600 UTC, and (d) 0600–1100 UTC on 4 November. Dots indicate mean potential temperature at the slope profile locations. Horizontal error bars denote the standard deviation of the linearly detrended time series for the different periods. Profiles are located northwest (SP_NW), north (SP_N), northeast (SP_NE) and south (SP_S ) of the city centre (Figure 1c)

Haid *et al*. (2020) showed that observations from T‐RH sensors installed along sloping terrain around Innsbruck (Figure 1c) can be used to construct pseudo‐vertical temperature profiles to analyse the stratification of the Inn Valley CAP. The calculation of potential temperature from T‐RH sensor data is described in Section 2.1. Figure 8a shows that the observed and simulated CAP is rather homogeneous along the Inn Valley during the onset of foehn in the Wipp Valley. LES‐dx40 exhibits a weak warm bias of up to 3 K compared to observations in the CAP below 1.2 km amsl. However, low‐level static stability is overall reasonably well reproduced in the simulation (Figure 8a).

While the observed CAP remains almost homogeneous along the Inn Valley until 1100 UTC on 4 November, LES‐dx40 develops a strong along‐valley heterogeneity with a shallower CAP from the city centre eastward (Figure 8b–d). Nevertheless, around midnight simulated potential temperatures at the bottom of the slope profiles are still similar. However, further aloft in the lowest 300 m agl, potential temperatures are up to 5 K lower at the western profile (SP_NW) compared to the others (Figure 8b). Lower mean potential temperatures at the Inn Valley floor west of SL74 and tilted mean isentropes near the surface are also depicted in Figure 7b,d,f.

During the remainder of the night, simulated mean potential temperature at the bottom of each slope profile increases except for the western profile (SP_NW, Figure 8b,c). Hence, the along‐valley asymmetry of the CAP increases during the night in LES‐dx40. Moreover, the simulated CAP is eroded at all profiles during the night (Figure 8b–d). Associated with this CAP thinning, the progression of turbulent mixing towards the valley floor is reflected in an increase in temperature fluctuations at lower levels (error bars in Figure 8). In contrast, no CAP thinning is observed during the first night of IOP 2 and the strongest temperature fluctuations stay elevated at the CAP–foehn interface (Figure 8b–d).

The simulated CAP also exhibits on average a weak heterogeneity in the cross‐valley direction. After midnight it is shallower near the slopes north and south of Innsbruck than at the valley centre (e.g., Figure 3f). The CAP thinning at the northern slope is caused by strong mixing (TKE in Figure 3f) and reversed (northerly) downslope flow associated with flow deflection (Figures 3e, 5a, b and 6a–c). The CAP thinning in the south is due to dynamic displacement by the foehn at the exit of the Wipp Valley (Figure 5a,b).

The continuous CAP warming during night‐time in LES‐dx40 leads to nearly mixed conditions and foehn breakthrough east of Innsbruck (SP_NE) soon after sunrise (cf. Figure 8c,d). During the morning, a shallow CAP only remains west of the city centre (Figures 5b, 7f and 8d). By about 1200 UTC on 4 November widespread foehn breakthrough has neutralized all profiles (cf. Figure 5c).

### 4.5 Shear flow instabilities below and above the foehn jet

LES‐dx40 indicates the presence of strong vertical wind shear and associated K‐H instability during several periods and in different regions. First of all, during the early phase of foehn onset in the Wipp Valley (1900–2230 UTC on 3 November), large‐amplitude K‐H waves can be found at the interface between the CAP reaching into northern part of the Wipp Valley and the foehn jet aloft. Figure 3g shows overturning isentropes and vertical wind speed exceeding 3 m·s^−1^ for a subdomain marked in Figure 3a. K‐H waves simulated in this shear layer have a wavelength of around 1.5 km. Hence, the diameter of a single wave crest, which causes a single large eddy after breaking, is about 800 m. This is larger than in lidar observations of eddies forming by shear‐flow instabilities further downstream at the foehn–CAP interface over the Inn Valley which suggest a diameter of around 400 m (Haid *et al*., 2020, their Figure 10). K‐H wave breaking in the forward (downstream) direction in Figure 3a,g induces turbulence. Total TKE averaged from 1900 to 2230 UTC on 3 November is mostly between 4 and 6 m^2^·s^−2^ in the shear layer below the foehn jet (cf. Figure 3a,b) with about 90% of the TKE being explicitly resolved in the LES. Turbulent mixing by K‐H wave breaking leads to cooling of the upper part and warming of the lower part of the shear layer (diverging mean isentropes south of EC_S in Figure 3b) and finally the formation of a nearly mixed layer until the flow reaches the Nordkette (vicinity of T4 in Figure 3b). With decreasing CAP depth, the turbulent foehn–CAP interaction zone descends too and finally disappears in the Wipp Valley but prevails in the Inn Valley until the morning of 4 November (Figure 3c,d).

In the final period of CAP removal (0600–1100 UTC on 4 November), breaking K‐H waves are still present and strongly deform the CAP. Figure 3i illustrates the distorted CAP with overturning isentropes for a subdomain in the Inn Valley indicated in Figure 3e. The amplitude of these waves is between 200 and 300 m and, therefore, slightly larger than the mean depth of the CAP (cf. Figures 3f and 7f). The maximum vertical wind speed is around 2 m·s^−2^. The horizontal wavelength and associated eddy diameter of the broken wave corresponds better to the previously mentioned lidar observations at the CAP–foehn interface in the Inn Valley of Haid *et al*. (2020). Mean total TKE in the shear layer on top of the CAP exceeds 10 m^2^·s^−2^ with about 80% of the strongest mean total TKE explicitly resolved by LES‐dx40 (Figure 3f).

Large‐amplitude breaking K‐H waves also form *above* the foehn jet with increasing foehn intensity. Figure 3h depicts the situation in a small elevated subdomain above EC_S in the Wipp Valley (Figure 3e). Here, K‐H wave breaking is illustrated by slightly backward‐leaning isentropes. The vertical wave amplitude is about 300–400 m and the horizontal wavelength about 2 km and therefore about twice the one in the Inn Valley (cf. Figure 3h,i). Vertical wind speed is slightly stronger than for the K‐H waves shown in Figure 3i. Breaking K‐H waves above the foehn jet contribute to an elevated secondary maximum of mean total TKE at around 1.5 km amsl in Figure 3f. These upper breaking K‐H waves contribute to the splitting of the capping inversion on top of the foehn flow. The inversion splitting eventually leads to the formation of a weakly stratified layer above the Inn Valley at about 1.5 km amsl compared to the pronounced inversion present southward of ELL between 1.7 and 2.0 km amsl (white contours in Figure 3e). However, above ELL a more stationary mountain wave can be found in LES‐dx40 (mean isentropes in Figure 3b,d,f) and MESO‐NUD (not shown) which appears to be excited over the adjacent mountain ridges and has radiated in the cross‐stream direction to the Wipp Valley centre by three‐dimensional wave dispersion (Zängl, 2003; Zängl and Gohm, 2006). Resolved turbulent processes in LES‐dx40 appear to be in line with observations that highlight the important role of small‐scale instabilities (Farmer and Armi, 2001). In numerical models that poorly resolve these processes (e.g., MESO‐NUD), mountain wave breaking appears to partly take over the role of K‐H wave breaking (Farmer and Armi, 2001).

**FIGURE 9:**
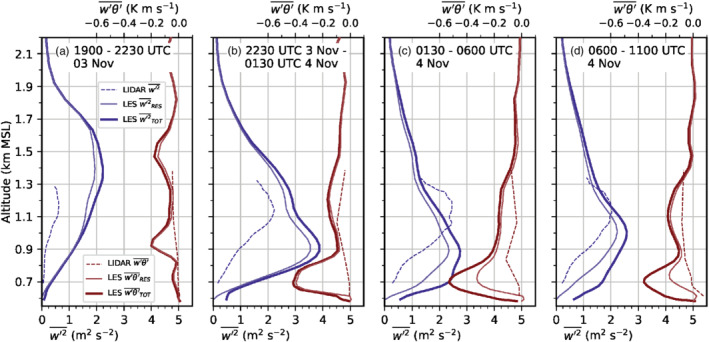
Mean vertical profiles of vertical velocity variance $\bar{w^{\prime 2}}$ (blue) and vertical turbulent sensible heat flux $\bar{w^{\prime }\theta^{\prime }}$ (red) for the periods (a) 1900–2230 UTC on 3 November, (b) 2230–0130 UTC, (c) 0130–0600 UTC, and (d) 0600–1100 UTC on 4 November near the city centre at the lidar site SL74 (Figure 1c). Vertical velocity variances and heat fluxes are estimated (parametrized) based on lidar observations (dashed lines). Total (TOT) and explicitly resolved (RES) variances and heat fluxes are derived from LES‐dx40m data and shown as thick (thin) solid lines. Corresponding subgrid‐scale (SGS) parts can be visually inferred from the difference between TOT and RES. LES‐dx40 profiles are based on a spatial average over the nine grid points closest to the lidar. Details on estimating heat fluxes from lidar data and temperature observations can be found in Haid *et al.* (2020)

Breaking K‐H waves are associated with intense vertical motions and therefore enhanced vertical velocity variance. LES‐ and lidar‐derived vertical velocity variances are shown in Figure 9 averaged for four periods at the lidar site SL74 near the city centre (Figure 1c). Figure 9a depicts the phase of foehn onset in the Wipp Valley in LES‐dx40. The maximum ${\bar{w^{\prime 2}}}_{\text{TOT}}$ is around 2.3 m^2^·s^−2^ for LES‐dx40. It occurs at about 1.4 km amsl near the level of maximum vertical wind shear and nearly neutral mean stratification (cf. Figure 3a,b), where K‐H instability below the foehn jet is simulated (Figure 3g). The simulated variance is at least three times higher than the observed variance averaged over this 2.5 hr period. However, the observed vertical velocity variance appears to be more intermittent and exhibits magnitudes comparable to the simulated ones for shorter 18 min periods (Haid *et al*., 2020, their Figure 7b). Except for the lowest 100 m agl, more than about 80% of the total vertical velocity variance (${\bar{w^{\prime 2}}}_{\text{TOT}}$) is explicitly resolved in the LES (${\bar{w^{\prime 2}}}_{\text{RES}}$). The remaining SGS part is only dominant close to the surface.

With the descending interaction zone between the Inn Valley CAP and the foehn jet during the night from 3 to 4 November, the maximum of ${\bar{w^{\prime 2}}}_{\text{TOT}}$ descends too and intensifies (Figure 9b,c). ${\bar{w^{\prime 2}}}_{\text{SGS}}$ near the surface from less than 0.1 to about 0.5 m^2^ (dot missing) s^−2^. In the late night, ${\bar{w^{\prime 2}}}_{\text{SGS}}$ shows a pronounced maximum of 1 m^2^·s^−2^ at around 700 m amsl which corresponds to about 40% of the total mean vertical velocity variance (Figure 9c). This maximum is collocated with the highest total mean TKE close to SL74 in Figure 7d. The agreement in the magnitude of observed and simulated vertical velocity variance is better for the second half of the night (Figure 9c) than for the first half (Figure 9a,b). Yet, the observed maximum is located about 200 to 300 m higher than the simulated one (Figure 9b,c), which is in agreement with the deeper observed CAP previously mentioned (Figure 8).

Although incoming short‐wave radiation is present after 0600 UTC on 4 November, near‐surface ${\bar{w^{\prime 2}}}_{\text{SGS}}$ (a proxy for surface‐driven convective mixing) increases only marginally in LES‐dx40 (Figure 9d). The maximum ${\bar{w^{\prime 2}}}_{\text{SGS}}$ is still located in the upper part of the simulated CAP at about 700 m amsl (cf. Figures 3f,i and 9c). Maximum ${\bar{w^{\prime 2}}}_{\text{TOT}}$ is around 2.5 m^2^·s^−2^ and located at 1,000 m amsl which is also the height of the strongest mean total TKE in Figure 3f. Observed and simulated variances during daytime (Figure 9d) are slightly lower than during night‐time (cf. Figure 9b,c).

Figure 9 illustrates that LES‐dx40 generally overestimates compared to observations the vertical velocity variance inside the valley, especially at low levels and during the early foehn stage. This may be evidence for too strong mixing by K‐H instability in the model and/or a deficiency in the timing of the foehn evolution. Below 800 m amsl, the influence of SGS mixing (i.e., ${\bar{w^{\prime 2}}}_{\text{SGS}}$) is low in the first part of the night (Figure 9a,b) but increases substantially afterwards (Figure 9c,d). It is noteworthy that the simulated *horizontal* velocity variances (not shown) are generally higher than ${\bar{w^{\prime 2}}}_{\text{TOT}}$. Mean horizontal velocity variances reach up to twice the magnitude of the vertical velocity variance of Figure 9. Nevertheless, the fraction of resolved and SGS variances is similar for both horizontal and vertical components.

Overall, the CAP erosion is too vigorous in the LES and leads to a strong along‐valley CAP heterogeneity and a too early foehn penetration to the Inn Valley floor compared to observations, as mentioned above. This appears to be caused by a too strong foehn–CAP interaction related to the resolved part of turbulence in LES‐dx40. Despite this discrepancy, LES‐dx40 provides valuable insight into microscale processes of CAP erosion for the following reason: The CAP structure in the LES during the *first* night of IOP 2 (3 to 4 November 2017) is very similar to the structure observed during the *second* night of IOP 2 (4 to 5 November 2017; Haid *et al*., 2020). More specifically, LES‐dx40 produces a heterogeneous CAP along the Inn Valley during the night with higher near‐surface potential temperatures in the east than in the west of the city of Innsbruck (e.g., Figures 5b and 8b,c) and the shallowest part of the CAP is present near the city centre (cf. Figure 7b,d). The same spatial structure has been observed during the second night of IOP 2 (e.g., figure 6 in Haid *et al*., 2020) and other IOPs (Muschinski *et al*., 2020). Moreover, the observed foehn breakthrough to the Inn Valley floor on the second day of IOP 2 occurred first in the east already before sunrise and a few hours later in the western part of the city (cf. observations at EC_W and EC_E in Figure 2e). This progression of the foehn breakthrough from west to east is also comparable to LES‐dx40 as described in Section 4.2. Therefore, LES‐dx40 does produce a realistic manifestation of a foehn–CAP interaction and we proceed with the analysis of the heat budget of the CAP based on result of LES‐dx40 to quantify the atmospheric processes responsible for the CAP erosion around Innsbruck.

## 5 HEAT BUDGET OF THE CAP IN THE LES

In this section, individual contributions to the mean total change of potential temperature in LES‐dx40 are used to investigate the processes of CAP erosion. Details regarding the derivation of these contributions can be found in Section 2.4 and Appendix B. Firstly, foehn onset in the Wipp Valley during the early night is discussed (Section 5.1). Subsequently, the remainder of the night is analysed, when the CAP in the Inn Valley is influenced by the foehn jet aloft (Section 5.2). Finally, the CAP removal in the Inn Valley after sunrise is investigated (Section 5.3).

### 5.1 Foehn onset in the Wipp Valley early in the night

**FIGURE 10:**
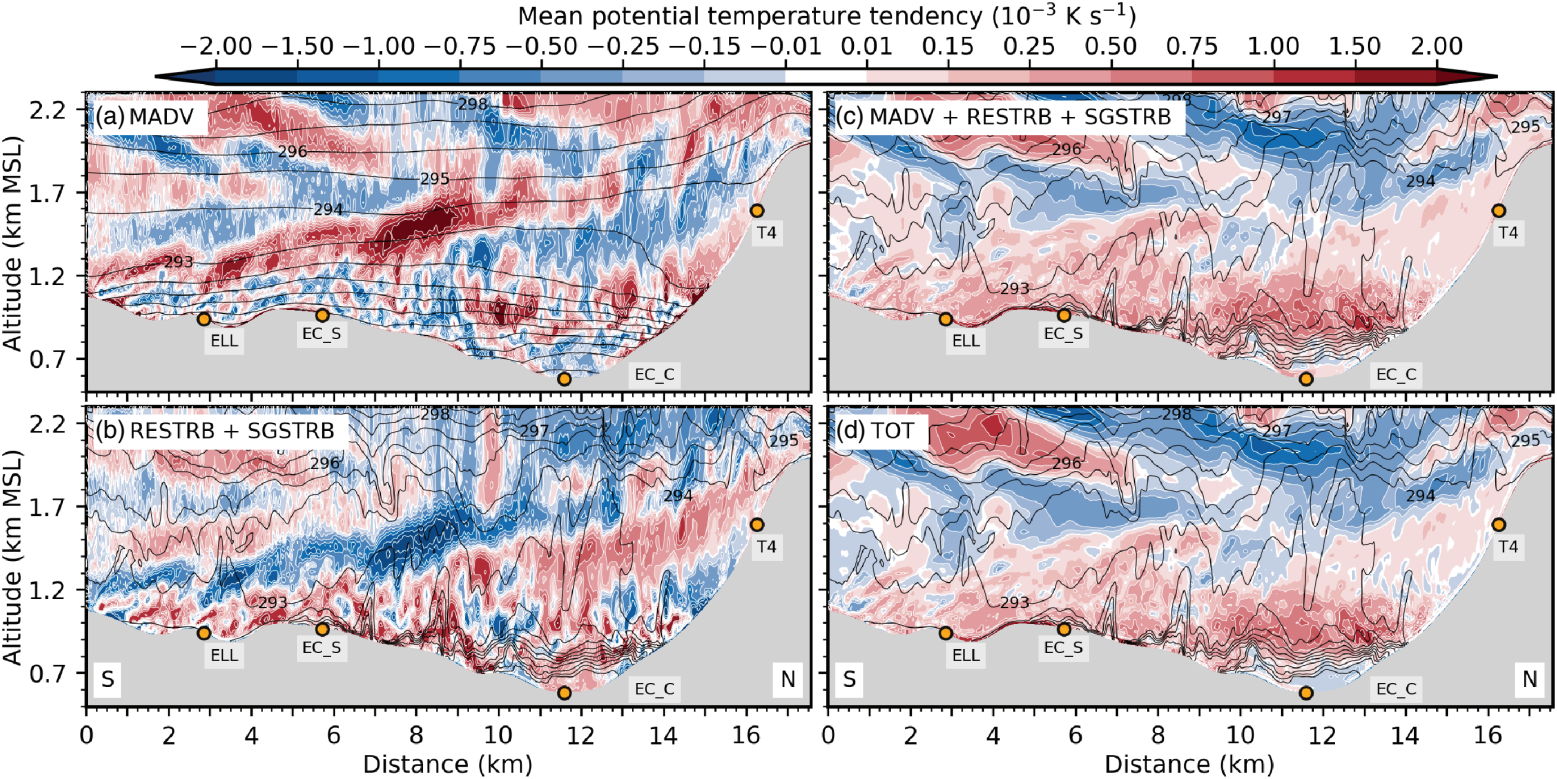
Components of the mean potential temperature tendency (colour shading, 10^−3^ K·s^−1^) along the transect WIPP (Figure 1c) based on LES‐dx40 data averaged over 1900–2230 UTC on 3 November due to (a) mean flow advection, (b) sum of resolved and SGS turbulence, (c) sum of (a) and (b), and (d) total tendency including radiative and microphysical processes. White contours of potential temperature tendency exceed the limits of the colour scale with intervals of 2 × 10^−3^ K·s^−2^. Black contours denote isentropes for (a) the above averaging period and (b–d) instantaneously at 2230 UTC on 3 November. Annotations and topography are as in Figure 3

As already mentioned in Section 4.1, the CAP in the Wipp Valley is essentially removed between 1900 and 2230 UTC on 3 November. The combined effect of advection and turbulent mixing (including K‐H wave breaking) warms the lower part of the atmosphere in the Wipp Valley (Section 4.5; Figure 10c). While heating due to turbulent mixing is mostly present in the lowest few hundred metres above the valley floor (Figure 10b), warm‐air advection with the mean flow dominates above the CAP (near the mean 293 K isentrope in Figure 10a). However, at this level, turbulent mixing leads to cooling (Figure 10b) and hence reduces the effect of advective warming (Figure 10c). The net effect leads to a nearly homogeneous layer between 0.8 and 1.5 km amsl with mean heating rates exceeding 0.25 × 10^−3^ K·s^−1^ (20 K·day^−1^) in the Wipp Valley and 0.75 × 10^−3^ K·s^−1^ (65 K·day^−1^) above the Inn Valley in Figure 10c. Comparing instantaneous isentropes at the end of the averaging interval in Figure 10b–d with the mean isentropes in Figure 10a shows that the CAP is eroded and replaced by a nearly mixed layer in the Wipp Valley while the CAP in the Inn Valley prevails, however with a reduced depth and increased stability. A stable stratification is present up to around 1 km amsl at 2230 UTC on 3 November in LES‐dx40 (Figure 10b–d).

**FIGURE 11:**
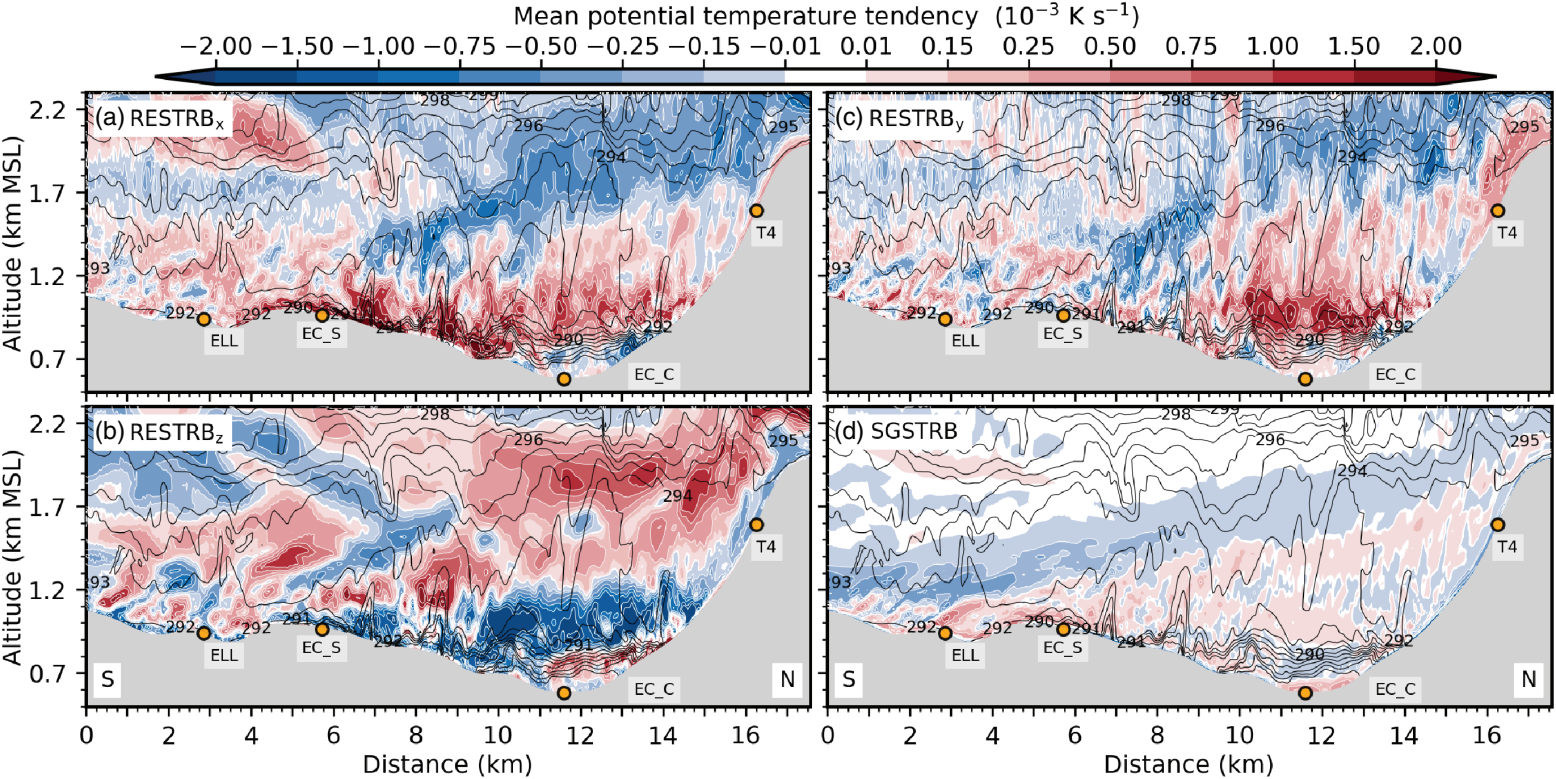
As Figure 10, but for mean potential temperature tendency due to the (a) west–east component, (b) vertical component, and (c) north–south component of resolved turbulence (RESTRB) and (d) total SGS turbulence. Black contours in all panels represent instantaneous isentropes at the end of the averaging period as in Figure 10b–d

Advective plus turbulent heating (Figure 10c) does not strongly differ from total heating (Figure 10d) in the Wipp Valley, which indicates that radiative and microphysical processes do not strongly affect the CAP erosion there. Within the Inn Valley, the sum of the mean flow advection and turbulent mixing on average mostly results in a warming near the valley floor and a cooling at about 0.7 km amsl (Figure 10c) with the latter mainly caused by mean flow advection with the west–east wind component (cf. Figure 10a–c). During this early period, microphysical and radiative processes cannot be neglected up to 200–300 m agl in the Inn Valley (cf. Figure 10c,d). The spurious formation of a shallow layer of low stratus at about 50–100 m agl west of SL74 leads to microphysical heating and cooling by radiative flux divergence (cf. simulated net radiation at EC_W in Figure 4b and Section 4.2). These effects are mostly compensating above 50 m agl. Cooling by evaporation of hydrometeors at the lowest two model levels overcompensates warming by SGS turbulent mixing near the surface (cf. Figures 10b–d and 11d). The exact reason for the formation of this very shallow layer of low stratus as well as the interconnection between the evaporation of hydrometeors and enhanced SGS turbulent mixing close to the surface is beyond the scope of this paper. However, this shows the influence of microphysical processes on the CAP formation in real‐case simulations, which is often not accounted for in idealized studies of CAP formation (e.g., Sheridan, 2019). However, contributions from microphysical processes to the CAP heat budget reduce during the night from 3 to 4 November and microphysics are considered rather negligible for the overall interaction between the Inn Valley CAP and the foehn flow in the LES.

Individual heating/cooling terms due to resolved and SGS turbulence are shown in Figure 11 and their sum in Figure 10b. Resolved turbulent vertical mixing warms the shallow CAP in the Inn Valley and cools a ∼500 m deep layer above (Figure 11b). On top of this cooling, starting at about 1.1 km amsl in the Wipp Valley, an inclined layer of heating by resolved vertical turbulent mixing extends northward towards the Nordkette. Most likely, this heating is the result of K‐H wave breaking underneath the foehn jet during the early stage when the CAP was deeper and extended into the Wipp Valley (cf. Figure 3a,g). SGS mixing has a similar effect to resolved vertical mixing inside the CAP, with warming near the surface and cooling above. However, the magnitude is much smaller – in places one order of magnitude – except near the surface where SGS turbulent mixing dominates (cf. Figure 11b,d). The above‐mentioned cooling by resolved vertical turbulent mixing on top of the Inn Valley CAP (Figure 11b) is mostly compensated by heating due to resolved horizontal mixing (Figure 11a, c). However, the combined effect shown in Figure 10b leads to a rather complicated and patchy field of mean turbulent heating and cooling in the Inn Valley above 0.9 km amsl.

### 5.2 Foehn–CAP interaction in the Inn Valley late in the night

**FIGURE 12:**
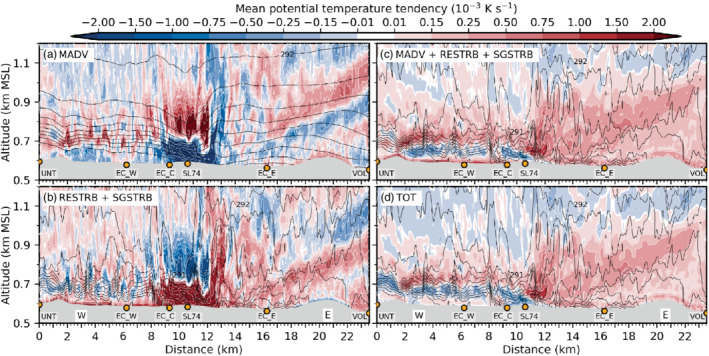
As Figure 10, but along the transect INN (Figure 1c) and averaged from 2230 UTC on 3 November to 0600 UTC on 4 November

**FIGURE 13:**
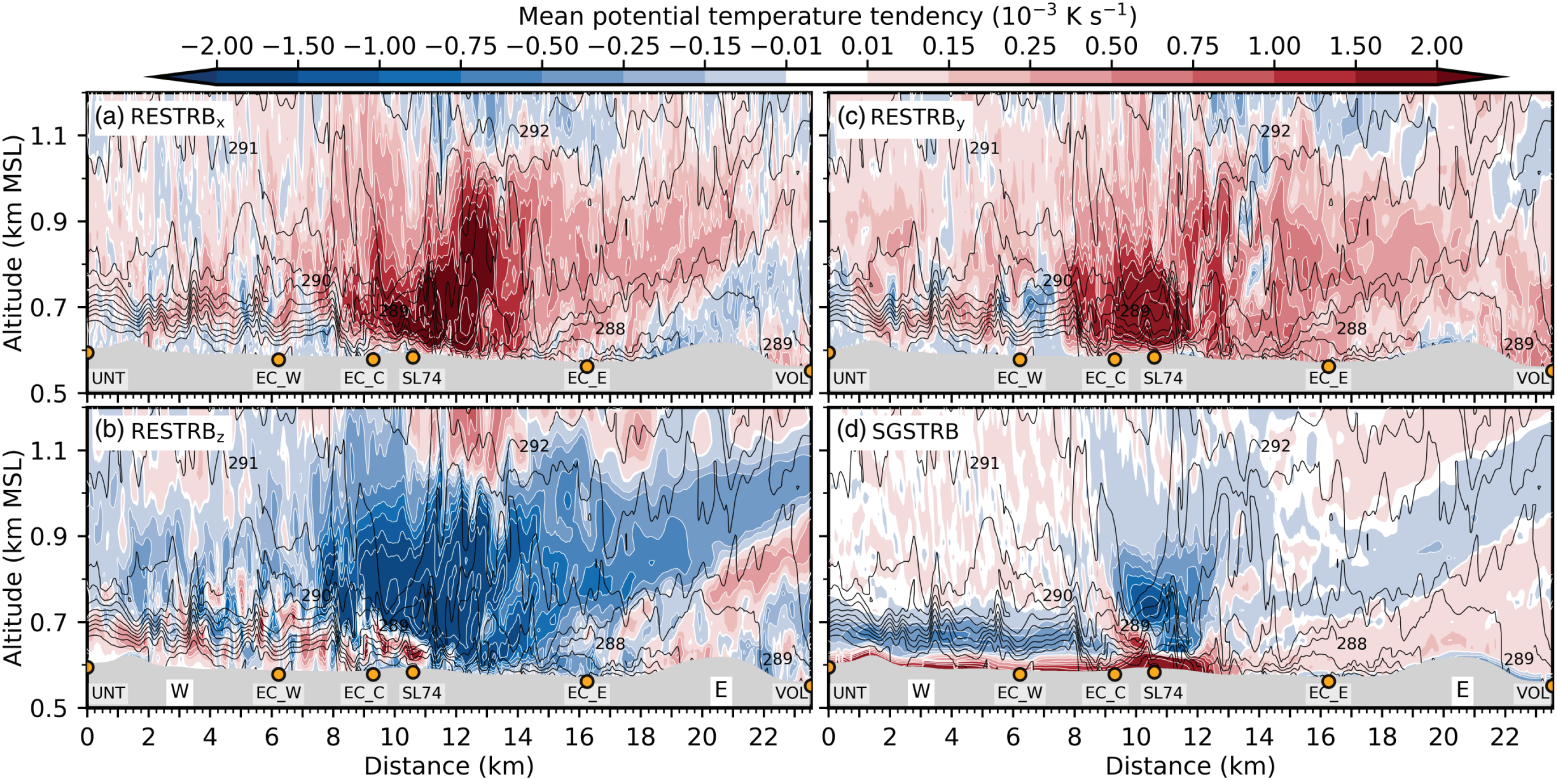
As Figure 11, but along the transect INN (Figure 1c) and averaged from 2230 UTC on 3 November to 0600 UTC on 4 November

For the remainder of the night from 3 to 4 November, we focus on the analysis of the CAP in the Inn Valley. Figure 12 shows the total and individual components of the potential temperature tendency averaged from 2230 UTC on 3 November to 0600 UTC on 4 November on a vertical cross‐section along the Inn Valley floor (INN in Figure 1c). The spatial distribution of *total* heating and cooling is very heterogeneous (Figure 12d). The valley atmosphere east of the city centre (i.e., east of SL74) is mostly affected by net heating, resulting in a nearly complete erosion of the CAP (Figure 12d). In contrast, the atmosphere above the city centre and further west exhibits a more complicated three‐layer structure of net heating/cooling (Figure 12d): heating in the upper part (700–900 m AMSL) and cooling in the lower part of the CAP (650–700 m AMSL) has intensified the capping inversion but also reduced the CAP depth (compare mean isentropes in Figure 12a with instantaneous isentropes at the end of the averaging period in Figure 12b–d). Heating by SGS turbulent mixing in the first 50 m agl decreases the stability below the capping inversion (cf. Figures 12c and 13d). Hence, the structure of the CAP has changed not only in the east but also in the west of the city during the night. However, a cooling mechanism has prevented a complete CAP erosion in the west.

**FIGURE 14:**
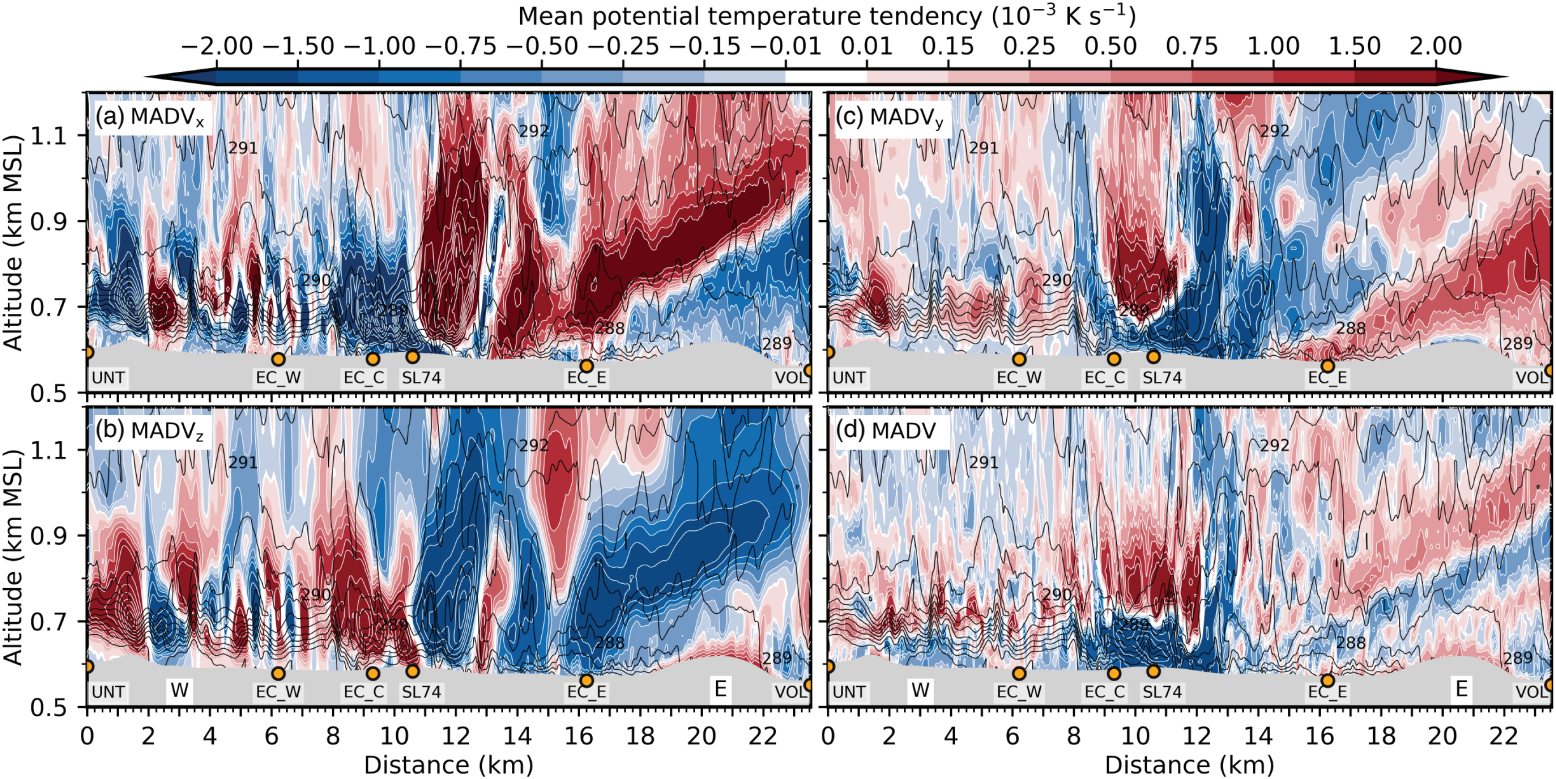
As Figure 12, but for the (a) west–east component, (b) vertical component, (c) the north–south component and (d) the sum of the mean flow potential temperature advection (MADV)

This cooling in the lower part of the CAP west of the city (i.e., below about 700 m amsl between UNT and SL74) is mostly due to cold‐air advection by pre‐foehn westerlies in the CAP (cf. Figures 12a and 14a) and partly due to turbulent mixing in the upper part of the CAP (Figure 12b). Although cold‐air advection is present near the surface, its effect is overcompensated by warming due to turbulent heat flux convergence (cf. Figure 12a–c). This near‐surface heating in Figure 12c is in turn reduced by radiative cooling near the valley floor (cf. Figure 12c,d). Microphysical processes contribute to cooling in the lowest 30 m agl (dissolving spurious low stratus) mostly before 0000 UTC on 4 November west of the city (Section 5.1).

Figure 13 illustrates individual contributions to heating/cooling by turbulent mixing. Within the CAP west of EC_C, SGS turbulent mixing is very important and partly exceeds the magnitude of the individual components of resolved mixing (cf. Figures 12b and 13). This illustrates that LES‐dx40 is not able to fully resolve turbulent processes in the stable boundary layer. Around and west of SL74, the effect of resolved vertical mixing results in a heating in the lower part of the CAP and a cooling above (Figure 13b). This change with height is caused by a change from vertical heat flux convergence to divergence at around 700 m amsl in Figure 9b,c.

Above the city centre (EC_C and SL74), magnitudes of the individual components of turbulent mixing and mean flow advection are largest but strongly compensating (Figures 13 and 14a–c). Most notably, strong cooling by resolved and SGS vertical turbulent mixing is reduced and partly compensated by resolved horizontal turbulent mixing (cf. Figure 13 and the sum in Figure 12b). However, this compensation is also present in other regions throughout the Inn Valley and not limited to the city centre.

Net cooling/heating by the sum of mean advection and turbulence above the city centre is about one order of magnitude smaller than the separate effect of advection and turbulence (compare values of about 0.5,× 10^−3^ K·s^−1^ in Figure 12c with values up to 8 ×  10^−3^ K·s^−1^ in Figure 12a,b). This implies that turbulence is mostly acting against advection in terms of heating/cooling above the city centre (cf. Figure 12a,b between 9 and 13 km on the abscissa). This is in line with findings of Vosper *et al*. (2013). Furthermore, separating turbulent mixing and mean flow advection into individual components (Figures 13 and 14a–c) illustrates that these components do not have equal (although similar) magnitudes and, more importantly, individual minima and maxima are not exactly opposite in phase. This makes it nearly impossible to deduce even the *sign* of the net effect, not to mention its magnitude, from single components.

### 5.3 CAP erosion in the Inn Valley during the morning of 4 November

**FIGURE 15:**
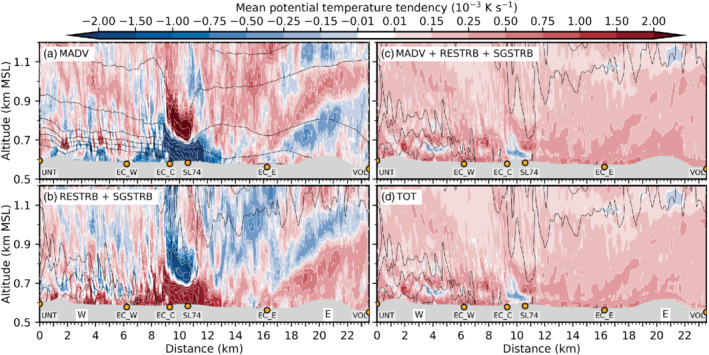
As Figure 12, but averaged over 0600–1100 UTC on 4 November

After sunrise at about 0600 UTC on 4 November, net radiation becomes positive after 0700 UTC in the simulation, in good agreement with observations (Figure 4). Similar to the situation during the late night (Section 5.2), strongest magnitudes of heating/cooling by advection with the mean flow and turbulent mixing are again present over the city centre around EC_C and SL74 and largely compensate each other (Figure 15a, b). Overall, the net heating of the Inn Valley atmosphere in the lowest 1 km agl is rather homogeneous (on average around 0.2 × 10^−3^ K·s^−1^ in Figure 15c). Hence, the above‐mentioned three‐layer structure of CAP heating/cooling west of the city centre disappears (cf. Figures 12 and 15). In this region, SGS turbulent mixing near the surface is slightly reduced compared to night‐time conditions, while the magnitudes of the individual components of resolved turbulent heating/cooling have generally increased in magnitude (doubling in some places) below 1 km amsl (not shown). Magnitudes of mean flow advection (mostly cooling below 0.7 km amsl) and turbulent mixing (mostly heating) have increased compared to nighttime (cf. Figures 12a,b and 15a,b). During the morning, increased heating by turbulent mixing up to 1 km amsl now fully compensates cooling by mean flow advection and leads to a net average heating effect west of the city centre. This is especially visible in the lowest 100 m agl (Figure 15c). Hence, until noon this net heating results in an almost complete CAP erosion and associated foehn breakthrough also west of the city (nearly mixed conditions represented by instantaneous isentropes in Figure 15b–d and Figure 5c).

East of the city centre, the CAP is nearly completely eroded during night‐time (cf. isentropes at 0600 UTC on 4 November in Figure 12b–d) and foehn intermittently already reaches the valley floor after 0600 UTC. The final erosion of the shallow CAP (about 50 to 100 m deep) east of the city centre near EC_E is caused by a combination of turbulent and advective heating (Figure 12a,b). This heating leads to a completely mixed layer until 1100 UTC on 4 November (instantaneous isentropes in Figure 12d).

## 6 DISCUSSION

### 6.1 CAP heterogeneity and pre‐foehn westerlies

**FIGURE 16:**
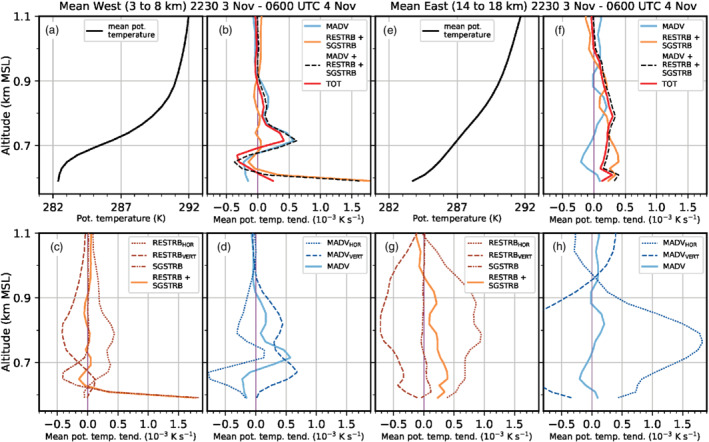
Mean vertical profiles based on LES‐dx40 data averaged from 2230 UTC on 3 November to 0600 UTC on 4 November shown in Figure 13 along the transect INN (Figure 1) and from (a–d) 4–8 km west of the city centre, and (e, f) 13–18 km east of the city centre. (a, e) show mean potential temperature (K). (b, f) show mean potential temperature tendencies (K·s^−1^) for the averaging interval resulting from mean flow advection (MADV), resolved and SGS turbulent mixing (RESTRB+SGSTRB), the sum of the former and the total effect including microphysical and radiative processes (TOT). (c, g) show individual mean potential temperature tendencies due to horizontal RESTRB, vertical RESTRB and SGSTRB and their sum. (d, h) show mean potential temperature tendencies due to horizontal and vertical MADV and their sum

Results in Section 3 and 4 have shown that the LES is superior to the mesoscale WRF simulation, but still predicts a too early foehn breakthrough in the Inn Valley. However, as mentioned above, the LES of the *first* night of IOP 2 exhibits a CAP structure which is very comparable to the observed CAP during the *second* night of IOP 2 (Haid *et al*., 2020) and other IOPs (Muschinski *et al*., 2020). Moreover, the schematic distribution of isentropes along the Inn Valley proposed by Haid *et al*. (2020, their figure 19b), with a depression over the city centre, is supported by LES‐dx40 in Figure 7b. The reason for this local CAP depression is strong shear‐induced turbulent mixing due to K‐H instability underneath the foehn jet that emanates from the Wipp Valley. This K‐H wave breaking is explicitly captured by LES‐dx40 (e.g., Figure 3) and has also been observed by multiple Doppler wind lidars (Haid *et al*., 2020). The associated vertical velocity variance and heat flux in the LES (about 3 to 4 m^2^·s^−2^ and −0.4 to −0.5 K·m^−1^·s^−1^ in Figure 9) are comparable in magnitude but slightly higher in absolute value than the observed ones for the first and second nights of IOP 2 (about 2 to 3 m^2^·s^−2^ and −0.1 to −0.2 K·m^−1^·s^−1^ in figures 16 and 17 of Haid *et al*., 2020).

Differences in the CAP evolution west and east of the city centre during night‐time are highlighted by mean vertical profiles of heating tendencies in Figure 16. These profiles represent horizontal averages along the cross‐sections shown in Figures 12, 13, 14, between 3 and 8 km (mean western profile; Figure 16a–d) and between 14 and 18 km on the abscissa (mean eastern profile; Figure 16e–h). Overall, temperature advection with the mean flow is mostly a cooling source in the lower part of the CAP (especially west of the city of Innsbruck) and a heating source in the upper part of the capping inversion in LES‐dx40 (Figure 16a–f). West of the city centre, this cooling is caused by horizontal advection with the pre‐foehn westerlies close to the surface (Figure 16d). Individual components of the resolved and SGS turbulent mixing compensate above 700 m amsl and result in a potential temperature tendency close to zero for the western profile (Figure 16c).

Close to the valley floor, SGS turbulent mixing dominates and leads to a strong heating at the surface and a weak cooling around 650 m amsl (Figure 16c). However, the mean total effect is only weakly positive at the surface for the western profile (Figure 16b). The difference between the dashed black and solid red lines in Figure 16b,f indicates the influence of microphysical and radiative processes. The above‐mentioned presence of spurious low stratus clouds below 700 m amsl around midnight west of the city leads to (a) slightly enhanced radiative cooling at around 700 m amsl (while it is decreased below, e.g., net radiation in Figure 4b), and (b) strong cooling in the lowest 30 m agl by evaporation of hydrometeors. The latter nearly compensates the strong heating by SGS turbulent mixing at the valley floor (cf. Figure 16b,c). With this compensating effect, the magnitude of the mean total potential temperature tendency at the valley floor is comparable to the lowest 50 m agl of the eastern profile (cf. Figure 16b,f) which is *not influenced* by the spurious low stratus formation. In the east, radiative cooling is the only additional contribution to the potential temperature tendency net effect and is strongest near the surface (Figure 16f). Therefore, the overall influence of the spurious low stratus on the foehn–CAP interaction is considered almost negligible. However, the interconnection and feedback between microphysical cooling and SGS turbulent mixing at the valley floor west of the city has not been investigated further as it is beyond the scope of this paper.

The total effect of all contributions exhibits a three‐layer pattern of net warming, cooling and again warming across the depth of the CAP west of the city centre which is dominated by mean flow advection, except for the lowest 30 m agl (Figure 16b). This three‐layer structure in the net temperature tendency has increased the stability at around 700 m amsl and decreased at the surface (Figure 16a). In contrast to the western profile, the eastern profile is characterized by a more homogeneous net heating up to 1 km amsl during night‐time (Figure 16f) which has resulted in the mentioned along‐valley CAP heterogeneity (Figure 12). This net heating is dominated by turbulent mixing (Figure 16f) and especially by its resolved horizontal component (Figure 16g).

Zängl and Gohm (2006) argue that the asymmetric deflection of the foehn jet at the Nordkette (i.e., stronger towards the lower Inn Valley; Figure 6) is caused by a smaller deflection angle towards the east. They state that the preferred eastward deflection tends to accelerate the foehn breakthrough to the east of Innsbruck compared to the western part of the city. However, they do not provide an analysis of the dominant heating mechanisms associated with this flow deflection. They refer to “deflection of warm air into the Inn Valley" which could be interpreted as warm air advection due to CAP displacement. The latter has been identified as an important process of CAP erosion in the Rhine Valley (Flamant *et al*., 2006). However, our heat budget analysis shows that the more dominant process of CAP erosion in the east of the city is turbulent mixing during the whole night (Figure 16f,h). While temperature advection with the mean flow represents a cooling process during large parts of the CAP erosion phase near the Inn Valley floor east of the city, it contributes only weakly to heating in the east in the upper part of the CAP (Figure 16h,f). However, we should notice that a clear partitioning into turbulent and mean advective heating is error‐prone in the case of rapid temperature changes within the averaging interval of 30 min used to derive these quantities (cf. Appendix B). For example, a rapid CAP displacement in this short period would most likely be depicted as turbulent rather than advective heating.

**FIGURE 17:**
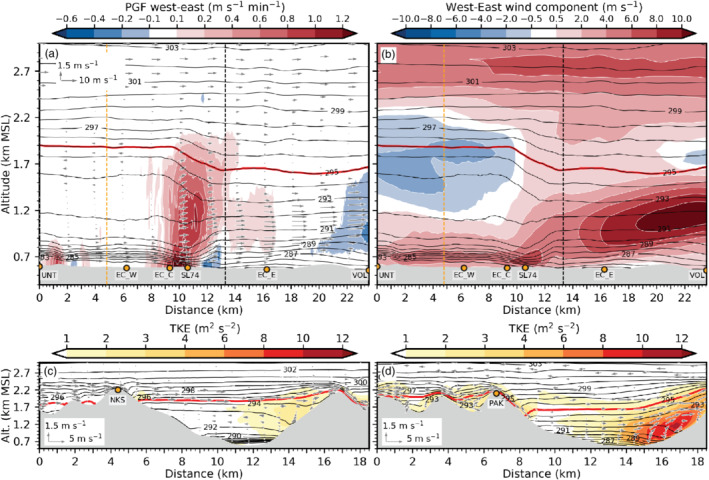
Vertical cross‐section of LES‐dx40 data averaged from 2230 UTC on 3 November to 0600 UTC on 4 November along the transect INN (Figure 1c) of (a) mean acceleration due to the west–east pressure gradient force (PGF, colour shading, m·s^−1^·min^−1^) and (b) mean west–east wind speed (colour shading, m·s^−1^). Vertical cross‐sections of mean total TKE (colour contours, m^2^·s^−2^) along the transects (c) NS1 and (d) NS2 across the Inn Valley (Figure 1c). Black lines denote mean isentropes with a 1 K interval. The 295 K mean isentrope is highlighted as thick red line. (a, c, d) show transect‐parallel mean wind vectors in grey. Note the different vector scaling between (a) and (c, d). The intersections of NS1 and NS2 with INN are indicated in (a, b) as dashed orange and black vertical lines, respectively

The along‐valley CAP heterogeneity which develops during nighttime appears to be not a unique feature of IOP 2, but has also been identified for other MAP and PIANO IOPs (e.g., Zängl and Gohm, 2006; Muschinski *et al*., 2020). Inside the CAP, the flow is characterized by pre‐foehn westerlies which are on average strongest near the city of Innsbruck (Figure 17b). According to Zängl (2003), these westerlies are the result of a general, weak down‐valley flow that is locally enhanced near the city. Two processes have been proposed to explain this local wind enhancement as a result of an intensified along‐valley pressure gradient: (I) A west–east asymmetry in the gravity‐wave field, with stronger wave‐induced subsidence and associated warming east of the city (to the lee of PAK; cf. Figure 1c) than west of the city (Zängl, 2003) and (II) a shallower and/or weaker CAP east of the city (Zängl and Gohm, 2006).

In order to test these two hypotheses, we compiled Figure 17 which represents conditions in the Inn Valley averaged over the period 2230 UTC on 3 November to 0600 UTC on 4 November. Figure 17a depcits the acceleration due to the along‐valley pressure gradient force (PGF) in relation to the potential temperature field. Pre‐foehn westerlies are present in the CAP west of SL74. Their maximum with winds exceeding 6 m·s^−1^ occurs near SL74 (Figure 17b) at the location of the strongest acceleration due to the along‐valley pressure gradient (PGF exceeding 1.2 m·s^−1^·min^−1^ near the surface in Figure 17a). While part of this pressure gradient is clearly caused by the along‐valley temperature heterogeneity *inside* the CAP, more than 60% of the total magnitude near the surface results from a horizontal temperature gradient *above* the CAP in the layer between 1.2 and 2.1 km amsl (cf. PGF exceeding 0.8 m·s^−1^·min^−1^ at 1 km amsl above SL74 in Figure 17a). This gradient is depicted by slanted isentropes above the city centre (highlighted 295 K isentrope in Figure 17a,b). The reason for this along‐valley temperature gradient in the upper part of the valley atmosphere is stronger foehn‐induced subsidence and associated adiabatic warming southeast of the city (leeside of PAK; Figure 17d) than southwest of Innsbruck (leeside of NKS; Figure 17c). This stronger mountain wave activity in the southeast is highlighted by the 295 K isentrope which subsides by about 300 to 400 m on the leeward side of PAK (Figure 17d) while it does not subside to the lee of NKS (Figure 17c). The stronger adiabatic warming downstream of PAK is also illustrated in Figure 6a–c. It is important to notice that the upper‐level pressure gradient due to gravity‐wave asymmetry superimposes onto the low‐level pressure gradient due to CAP heterogeneity and enhances the total pressure gradient inside the CAP. Therefore, in our simulation, enhanced pre‐foehn westerlies in the city centre inside the CAP are caused by a superposition of processes (I) and (II) proposed by Zängl (2003) and Zängl and Gohm (2006). This conclusion is supported by results from Muschinski *et al*. (2020), who showed for IOP 2 that the pure hydrostatic effect of CAP heterogeneity on the near‐surface pressure gradient could only explain the observed pressure gradient by assuming a rather deep CAP of 300 m (figure 8a in Muschinski *et al*., 2020), whereas it was shallower especially on the second day of IOP 2 (figure 6b in Haid *et al*., 2020). In other words, the observed total pressure gradient must be influenced by additional temperature heterogeneity above the CAP which most likely resulted from gravity waves.

The local CAP depression above the city centre with a width of about 1 to 3 km (Figures 7 and 17a,b) is caused by the interaction of the southerly foehn jet from the Wipp Valley with the CAP near the valley exit. Zängl and Gohm (2006) have shown that upstream (i.e., blocking) effects of the Nordkette mountain range north of Innsbruck reduce the flow speed of the foehn exiting the Wipp Valley. They argue that this in turn retards the CAP erosion by turbulent vertical mixing above the city. However, our results illustrate that, in terms of TKE, turbulent mixing is strongest above Innsbruck downstream of the Wipp Valley exit (Figures 7b,d,f and 3b,d,f). Hence, despite this deceleration and the associated lifting of the foehn jet above Innsbruck (Figure 7a,c,e), turbulent mixing by K‐H wave breaking constitutes an important heating process of the CAP (Figures 10b and 12b) contributing to the local decrease in CAP depth (Figures 7 and 17a,b). Above the city centre, heating by turbulent mixing is stronger than further east and west, where only the deflected foehn jet interacts with the CAP. The reason why the CAP is still eroded slower above the city centre than further east is the strong compensation of turbulent heating by cold‐air advection due to stronger pre‐foehn westerlies at the local CAP depression (Figure 12a). Numerical simulations by Zängl and Gohm (2006) with a horizontal grid spacing of about Δx = 500 m cannot fully resolve such a localized feature.

### 6.2 Foehn breakthrough in the Inn Valley

As illustrated in the previous section, the CAP in the LES is shallowest in the centre of Innsbruck during the first part of the night. However, during the second part of the night, the CAP erosion is stronger in the east of the city and leads to an earlier foehn breakthrough there than in the centre and west of the city (cf. Figures 5, 12 and 15). The eastward deflected foehn jet weakens the CAP east of the city mainly by turbulent erosion during the night (cf. Section 6.1 and Figure 16f). CAP displacement is negligible in the simulation and restricted to the northern foehn–CAP boundary at the slopes of the Nordkette (cf., Figure 5b between SL74 and EC_E and Figure 17d), which becomes visible as regular temperature fluctuations at some of the slope profile sites in LES‐dx40 (not shown).

Despite the discrepancies between LES‐dx40 and observations in terms of foehn evolution on the first day of IOP 2, the simulated foehn breakthrough on the first day has some similarities to the observed breakthrough on the second day of IOP 2 (Haid *et al*., 2020) and also to other PIANO and MAP IOPs (Zängl and Gohm, 2006; Muschinski *et al*., 2020). Foehn onset at the floor of the Inn Valley occurs first in the east of Innsbruck and later in the centre and west of the city. More specifically, on the second day of IOP 2 (Haid *et al*., 2020), and the first day of IOP 2 in the LES, it occurs first in the northeast. Haid *et al*. (2020) speculate that observed temperature fluctuations at the slope of the Nordkette are caused by K‐H waves propagating on the top of the CAP across the valley, amplifying and finally breaking at the mountain slope. Such amplifying and breaking K‐H waves occur in the LES and lead to strong CAP distortion and enhanced turbulent heating near the slope (cf. Figures 3i).

**FIGURE 18:**
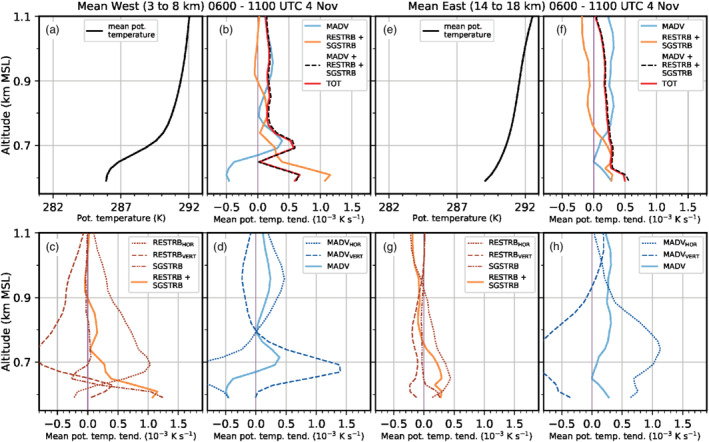
As Figure 16, but averaged over 0600–1100 UTC on 4 November

This raises the question of which process leads to the final erosion of the CAP and the complete foehn breakthrough in the city centre and further west after 1100 UTC on 4 November (cf. Figure 5c). Figure 18 shows averaged vertical profiles of mean potential temperature and mean potential temperature tendencies for the final phase of the CAP erosion in the west and east of the city averaged from 0600 to 1100 UTC on 4 November. East of the city, the net heating exhibits a similar vertical structure to during the night (cf. Figures 16f and 18f). Inside the weak and shallow CAP, most of this heating comes still from turbulent mixing (Figure 18g) with some additional contribution from horizontal advection (Figure 18h). In contrast, west of the city, the magnitudes of heating/cooling by advection and turbulent mixing are substantially larger than during night‐time except for SGS mixing near the surface (cf. Figures 16c,d and 18c,d). The resolved part of turbulent heating now contributes to heating up to 0.9 km amsl in Figure 18c. Although cold‐air advection with the mean flow close to the valley floor has also increased (cf. Figures16d and 18d), it does not overcompensate turbulent heating anymore, and therefore can no longer prevent CAP erosion in the west (cf. Figures16b and 18b). The increase of turbulent mixing west of the city centre is related to a stronger westward deflection of the foehn flow during daytime than during night‐time (Figure 6). During the night, most of the foehn jet is deflected eastward (Figure 6a–c) and leads to an ongoing erosion of the CAP there, and only a minor part is deflected up‐valley. Moreover, the eastward deflected foehn branch is located closer to the valley floor (and therefore the CAP) than the westward branch during the night (cf., Figure 17b). During the morning, the westward deflected foehn branch intensifies (Figure 6d,e) and more strongly interacts with the CAP west of the city. The stronger flow deflection towards the west is most likely associated with an increase in foehn wind speed in the Wipp Valley after 0600 UTC on 4 November due to the transition from shallow to deep foehn (Figures 2 and 3c,e).

Haid *et al*. (2020) state that the effect of solar radiation on the CAP destruction was negligible on the second day of IOP 2 but was more important for the intermittent foehn penetration to the valley floor on the first day. In the LES, solar radiation plays only a minor role for the final CAP erosion on the simulated first day of IOP 2, which further supports the comparison of the *simulated* first day with the *observed* second day of IOP 2. Surface sensible heat fluxes become hardly positive at EC_W (Figure 4b) and reach only 40 to 80 W·m^−2^ at other locations west of the city centre (not shown). Convective heating induced by positive surface sensible heat flux would be mainly represented in the LES by SGS turbulent heating close to the surface. The latter is even slightly smaller during the morning than during the night (cf. Figures 16c and 18c). However, the enhanced nighttime SGS mixing close to the surface in the west is also hypothesized to be interconnected with spurious microphysical processes near the valley floor, as mentioned in Section 6.1.

Some additional differences between observations on the second day and the simulation of the first day of IOP 2 are worth mentioning. In the city centre, the observed foehn breakthrough results in a *deflected* foehn on the second day (Haid *et al*., 2020) whereas it leads to a *direct* foehn on the first day in the LES (see above). Haid *et al*. (2020) link this deflected foehn, characterized by near‐surface reversed winds in the city centre, to an atmospheric rotor. However, consistent with direct foehn, no rotor‐like circulation forms in the LES. It is important to note that the occurrence of low‐level rotors depends, among other factors, on the gravity‐wave pattern aloft. Since the latter exhibits limited predictability (e.g., Doyle and Reynolds, 2008; Reinecke and Durran, 2009; Doyle *et al*., 2011), the predictability of the rotor, and hence the low‐level foehn type, is low also. Haid *et al*. (2020) conclude that CAP displacement in the east of the city by the eastward deflected foehn flow was an important process on the second day. In the LES, as mentioned above, turbulent CAP erosion, mostly due to horizontal mixing, is the dominant mechanism during the night in the east. However, these two statements are not necessarily in contradiction since Haid *et al*. (2020) refer to the final stage when the CAP was already very shallow, hence turbulent erosion could still have been important during the earlier stage.

Our results show that turbulent CAP erosion is more important than previously proposed (e.g., Lee *et al*., 1989; Zhong *et al*., 2003; Flamant *et al*., 2006). However, we believe that its importance strongly depends on the (valley) terrain geometry, specifically on the downstream topography. A boundless cold‐air reservoir above a plain may resist erosion more easily than a smaller volume of cold air in a valley, in which the deflected and hence channelled foehn jet can interact with the CAP over a larger distance (e.g., Zängl and Gohm, 2006).

### 6.3 Comparison of simulated and observed CAP heat budget

We now compare the simulated heat fluxes and potential temperature tendencies in the CAP to observations presented in (Haid *et al*., 2020). For IOP 2, Haid *et al*. (2020) estimated the vertical turbulent heat flux based on mean vertical temperature gradients and the eddy diffusivity calculated from lidar‐derived vertical velocity variance (assuming isotropic turbulence) by applying the Deardorff (1980) parametrization. The observed fluxes in Figure 9 are based on the same method. Furthermore, Haid *et al*. (2020) estimated the horizontal and vertical temperature advection from lidar‐derived mean wind profiles and temperature gradients between the slope profile stations indicated in Figure 1c.

**FIGURE C1:**
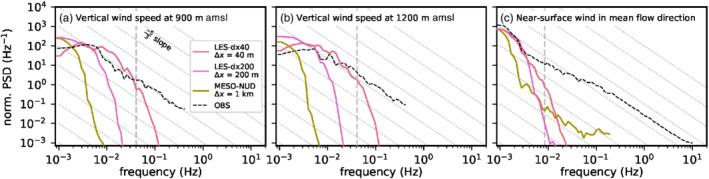
Averaged power spectral density (PSD) for the vertical wind speed near (a) 900 m and (b) 1,200 m amsl and (c) the horizontal wind component along the mean wind direction near the surface derived from observations (black dashed line) and WRF simulations MESO‐NUD, LES‐dx200 and LES‐dx40 (coloured lines). Observations are taken from (a, b) the SL74 lidar and from (c) EC_W. WRF model data are from the nearest grid point to those locations. Individual PSDs are normalized by the variance of the respective wind component and averaged for the period from 2230 UTC on 3 November to 0600 UTC on 4 November. Grey dotted lines indicate the ‐5/3 slope in the inertial subrange. The vertical dashed line indicates the effective resolution of LES‐dx40

During the first night, the magnitude of the simulated negative vertical heat flux in the CAP is up to five times larger than the estimated one based on observations (Figure 9). This is consistent with a more rapid CAP erosion in LES‐dx40. Interestingly, the same is even true when comparing the simulated fluxes of the first night (Figure 9) with the observed fluxes of the second night (Haid *et al*., 2020, their figure 17), for which the simulated and observed CAP depth and intensity are more similar and hence a better agreement in terms of fluxes would be assumed. Since this is not the case, the question arises whether the true vertical heat flux is systematically underestimated by the observations (e.g., due to the isotropy assumption) or overestimated by the LES (e.g., due to insufficient model resolution which may cause too vigorous mixing on the smallest resolvable scale). Unfortunately, we cannot answer this question conclusively without performing simulations with much higher resolution, which would be computationally very demanding. However, some support for the hypothesis of overestimated heat fluxes in the LES comes from the spectral analysis in Figure C1. Firstly, the simulated energy peak in the LES‐dx40 is close to the effective model resolution, which may point to energy accumulation there (Figure C1a,b). Secondly, the upper part of the inertial subrange, and hence the start of the energy cascade process, is not well captured. According to Cuxart (2015), such a simulation should rather be called “very large eddy simulation (VLES)”. Cuxart (2015) also notices that, for flows in a valley, a spectrum without inertial subrange (such as in the case of our LES) could result from a superposition of several spectra, each representing a different valley flow structure with a different scale. In contrast, observations indicate the existence of an inertial subrange (Figure C1).

The larger simulated than observed negative heat flux is in line with larger vertical velocity variances, especially during the early night (Figure 9). However, the relative difference is smaller and nearly vanishes during the second part of the night and morning when both observations and simulations indicate values between 3 and 4 m^2^ s^−2^. Nevertheless, simulated horizontal velocity variances are substantially larger (between 50 to 100% ) than the vertical velocity variance, indicating anisotropic turbulence. Strong anisotropic turbulence in breaking gravity waves during foehn was also found in Jiang and Doyle (2004). This would further emphasize the limitations of the isotropy assumption applied in Haid *et al*. (2020) ($\bar{u^{\prime 2}}=\bar{v^{\prime 2}}=\bar{w^{\prime 2}}$, with $\bar{w^{\prime 2}}$ explicitly measured) and point to underestimated vertical heat flux estimates based on observations. Despite these discrepancies, the simulated (Figure 9b–d) and estimated (Haid *et al*., 2020, their figures 16 and 17) vertical heat flux profiles exhibit a strong similarity. The magnitude of the negative heat flux increases with height in the lower part of the CAP (flux convergence) and decreases above (flux divergence). This results in heating (cooling) due to vertical mixing in the lower (upper) CAP. When assuming anisotropic turbulence in the form of $\bar{u^{\prime 2}}=\bar{v^{\prime 2}}=2\bar{w^{\prime 2}}$ with the method of Haid *et al*. (2020), strongest magnitudes of the estimated vertical heat flux would increase to around −0.2 K·m^−1^·s^−1^ in Figure 9 and better fit the simulated values in the layer between 0.9 and 1.3 km amsl. However, near the surface (below 0.9 km amsl), vertical heat fluxes in LES‐dx40 are still largely overestimated in Figure 9b–d.

The role of horizontal mixing could not be evaluated in Haid *et al*. (2020) due to the lack of appropriate measurements. However, our LES‐dx40 indicates that the contribution of horizontal mixing to the heat budget is crucial, especially during the final phase of CAP erosion. Potential temperature tendencies due to horizontal and vertical mixing have the same order of magnitude but often the opposite sign, indicating partial compensation of the two effects (Figures 11 and 13; Figures 16c and 18c). Hence, the net effect of turbulent mixing can be substantially smaller than those due to individual components. As mentioned above, cooling by resolved vertical turbulent mixing in the upper part of the CAP and mean flow advection is overcompensated between 100–300 m agl by resolved horizontal turbulent heating, leading to the nearly complete erosion of the CAP in the west.

Haid *et al*. (2020) found that, for the second day of IOP 2, heating by vertical turbulent mixing within the CAP (while neglecting the role of horizontal mixing) was compensated by cold‐air advection. The latter was mainly the result of strong along‐valley CAP heterogeneity combined with pre‐foehn westerlies. Haid *et al*. (2020) speculate that cold‐air advection was even overcompensating the effect of turbulent mixing since the net temperature tendency was negative (i.e., net cooling) for the analysed night‐time period. However, since the two horizontal advection components (based on temperature gradients measured over a horizontal distance of about 2.5 km) were more than one order of magnitude larger than vertical turbulent heating, the question arose whether these coarse‐resolution advection estimates are representative for the measurement site. Indeed, LES data for the first day in Figure 14 shows that individual advection components are much larger than individual components of turbulent mixing (cf., Figure 13), especially near the city centre and west of it. Absolute values of advective heating/cooling in Figure 14 reach up to about 0.018 K·s^−1^ (1500 K·day^−1^), which is comparable to the estimates of Haid *et al*. (2020), whereas corresponding horizontal and vertical turbulent heating reach about 0.008 K·s^−1^ (700 K·day^−1^; Figure 13). Moreover, Figure 14a,b and Figures 16d and 18d show that horizontal advection, especially along the valley, is partly compensated by vertical advection and hence the net effect of mean flow advection is usually weaker and more homogeneous (Figure 14d). Last but not least, near the city centre at about SL74, where the CAP is shallowest, the along‐valley advection in Figure 14a changes sign from cooling in the west to heating in the east. Both results, i.e., partial compensation and sign change, are in line with the findings of Haid *et al*. (2020).

## 7 CONCLUSIONS

A large‐eddy simulation (LES) was performed of a south foehn event that occurred between 3 and 5 November 2017 during IOP 2 of the PIANO field experiment in the Inn Valley near the city of Innsbruck (Austria). The goal was to improve the understanding of cold‐air pool (CAP) erosion due to foehn–CAP interaction and the associated breakthrough of foehn at the valley floor. More specifically, the role of different processes contributing to the CAP heat budget, especially turbulent and advective heating, were assessed. This modelling study complements a detailed observational study of the same event by Haid *et al*. (2020). The key findings are:

- With respect to CAP evolution and structure, the LES (Δx=40 m) proves to be superior to a mesoscale simulation (Δx=1 km) which provides the initial and boundary conditions for the LES. Yet, the foehn breakthrough in the Inn Valley occurs still too early in the LES compared to the observations and this may be related to still insufficient model resolution. Nevertheless, the simulated *first* day of IOP 2 is very similar to the observed *second* day. This similarity justifies a detailed analysis of CAP structure and erosion on the simulated first day and a comparison with observations from the second day.
- During night‐time, the CAP in the west–east aligned Inn Valley near the exit of the south–north oriented Wipp Valley exhibits strong heterogeneity near Innsbruck. This has also been observed during other IOPs of the PIANO project (Muschinski *et al*., 2020). West of the city, the CAP exhibits a pronounced capping inversion which is not present to the east where near‐surface temperatures are also higher. Moreover, the CAP exhibits a local depression near the city centre that is associated with the strongest westerly (downvalley) flow in the CAP. The local maximum of these so‐called pre‐foehn westerlies is the result of a local maximum of the along‐valley pressure gradient caused by a superposition of two effects proposed by Zängl (2003) and Zängl and Gohm (2006): along‐valley CAP heterogeneity in the lower part and gravity‐wave asymmetry in the upper part of the valley atmosphere. This is in line with findings of Muschinski *et al*. (2020).
- *East of the city*, CAP erosion by turbulent mixing is stronger than to the west and already leads to a weaker CAP during night‐time. The turbulent heating results from turbulent interaction between the CAP and the foehn jet, the latter deflected by the mountain range north of Innsbruck preferentially in the downvalley (eastward) direction. Furthermore, the total effect of temperature advection is less important in the east. Deflection of the foehn jet by the mountain range north of Innsbruck has already been found in previous studies, while its relation to the turbulent erosion of the CAP east of the city centre has not been investigated until now.
- The reason for the stronger CAP to the *west of the city* during IOP 2 is twofold. Firstly, the westward deflected foehn branch is weaker during night‐time, which implies weaker turbulent erosion. Secondly, turbulent heating is more strongly compensated by cold‐air advection due to pre‐foehn westerlies. However, the vertical structure of advective and turbulent heating/cooling in and above the CAP can be rather complicated and phase shifted. This results in a strongly height‐dependent net heating/cooling that changes the CAP structure in time, for example by strengthening the capping inversion and neutralizing the CAP near the surface. The ultimate CAP erosion in the west is caused by enhanced turbulent erosion when the westward deflected foehn branch intensifies due to a transition from shallow to deep foehn during IOP 2.
- The local CAP depression in the *centre of the city* is caused by its proximity to the exit of the Wipp Valley. In this area, turbulent heating due to the foehn jet emanating from the Wipp Valley is strongest. However, this local CAP depression also leads to the strongest advective cooling which largely compensates turbulent heating. Consequently, CAP erosion is slower near the city centre than to the east of Innsbruck during IOP 2.
- In contrast to previous studies, shear‐induced turbulence related to Kelvin–Helmholtz (K‐H) instability proves to be an important CAP erosion process. It is important not only at the foehn–CAP interface but also at the top of the foehn jet. There, K‐H wave breaking in the LES appears to take over the role of mountain wave breaking usually simulated by mesoscale models in forming a mixed layer on top of the downslope windstorm. However, it remains to be seen whether structure and magnitude of K‐H wave breaking fundamentally changes with a further increase in model resolution.
- Radiative and microphysical processes are less important during the foehn–CAP interaction in this specific case in the LES. However, other studies indicate that the former is important in other foehn events and may even be the dominant process. Yet, a quantitative estimate of bottom‐up heating for such events is still missing. Microphysical processes are mostly negligible in the LES, except for a period around midnight with spurious low stratus formation close to the valley floor west of the city of Innsbruck. However, the reason for this hydrometeor formation has not been investigated thoroughly and is beyond the scope of this study.
- Horizontal and vertical components of turbulent and advective heating are strongly variable in time and space, often have opposite sign, and may easily be one order of magnitude larger than net heating, especially for strong winds in complex terrain. Moreover, horizontal components can be much stronger than vertical components. This poses a great challenge for current observational systems for the following two reasons. Firstly, they often provide point measurements and, hence lack representativeness to capture spatial heterogeneity. Secondly, they usually do not capture all components with the same accuracy, and some of them not at all. Consequently, they inherently fail to close the heat budget in such conditions. Nonetheless, they are undoubtedly necessary to challenge the quality of the numerical models. Therefore, the current solution to this dilemma must be a synergistic approach, in which observations are used to approve the quality of these models and then use these (yet imperfect) models to close the gaps in observational datasets. But even here the challenge remains that the set‐ups of both observational and modelling systems need to be carefully chosen for each phenomenon of interest to minimize relevant errors.

Future work should (a) extend this heat budget analysis to other foehn events to clarify the role of additional processes such as bottom‐up heating, (b) investigate whether mesoscale simulations systematically overestimate CAP erosion compared to LES and, if so, try to assess whether or not this is related to deficiencies in the boundary‐layer parametrization, and (c) more closely investigate the value of simulations with a hectometre‐sized grid in bridging the gap between the mesoscale and the high‐resolution LES. Moreover, it would be worth assessing whether the improvement in representing CAP erosion processes by a further increase in LES resolution, say by a factor of five, justifies the enormous increase in the associated computational costs. Ultimately, these efforts may help to improve limited‐area models applied to numerical weather prediction in complex terrain.

## A OBSERVATION NUDGING

Data from automatic weather stations (AWSs) at locations in the valleys below 1,200 m amsl (Figure A1a) are used in the MESO‐NUD simulation for observation nudging (Liu *et al*., 2006; Liu *et al*., 2008) from lead time 0 to +6 hr. The AWSs are operated by the Austrian weather service (ZAMG), the hydrographic service of Tyrol, the weather service of the autonomous province of South Tyrol and the Department of Atmospheric and Cryospheric Sciences of the University of Innsbruck (ACINN). From these AWSs, 10‐min averaged values are used. Observations above 1,200 m amsl including radiosonde data are not used since their spatial density is too low and would introduce model artefacts in the complex terrain around Innsbruck. Observation nudging is active for temperature and moisture with a nudging coefficient of 4 × 10^−3^ s^−1^ and a time window of 16 min. However, the nudging coefficient is ramped up (down) from (to) zero in the first (last) quarter of the time window and therefore nudging is only fully active for a time window of 8 min around the time of the respective observation. Horizontal weighting of observations uses the MM5 scheme (namelist variable obs_sfc_scheme_horiz = 1) with a horizontal radius of influence of 10 km. In the vertical, the nudging coefficient is constant up to 50 m agl and ramped down to zero at 200 m agl for temperature and moisture and all stability regimes using obs_sfc_scheme_vert = 0. These settings are chosen to reproduce the CAP at 1800 UTC on 3 November as close to reality as possible for the initialization of the LES.

The difference in potential temperature on the lowest model level (10 m agl) at 1800 UTC on 3 November (lead time +6 hr) between the simulation with (MESO‐NUD) and without (MESO‐DEF) nudging is shown in Figure A1a. The strongest differences occur south ot the main Alpine crest, where MESO‐NUD is more than 8 K colder than MESO‐DEF, while differences in the Inn Valley and around the city of Innsbruck are up to 5 K. There are only a few random spots where MESO‐NUD is 1 to 2 K warmer than MESO‐DEF. The mean model bias of potential temperature at all AWSs north and south of the main Alpine crest is shown in Figure A1b and c, respectively. MESO‐DEF has a mean bias at lead time +6 hr of about 2 K in both regions. This bias is close to zero in MESO‐NUD. Hence, data assimilation via observation nudging improves the formation of the CAP in the Alpine valleys within the first 6 hr of simulation. Lead time +6 hr represents the starting time of LES‐dx40 and LES‐dx200. However, the error reduction in MESO‐NUD is not sustainable. The mean error quickly increases after the nudging period and reaches about the same value as MESO‐DEF at lead time +18 hr.

## ONLINE CALCULATION OF LES DIAGNOSTICS AND OTHER MODEL CODE MODIFICATIONS

### 1

As described in Section 2.4, the source code of the WRF model (version 4.1) is extended for the online calculation of mean and turbulent quantities during model integration. These quantities include resolved and subgrid‐scale (SGS) velocity variances and covariances, resolved and SGS heat fluxes, as well as tendency terms for the potential temperature and the acceleration of horizontal wind components $\tilde{u}$ and $\tilde{v}$ due to pressure gradient force. The modified WRF source code is available at Zonedo (Umek, 2020).

The time‐averaging operator of Equation (1) is implemented in the WRF model by a modification of the already available time averaging of mass‐coupled advective velocity components in module_avgfluxes.F (activated via namelist variable do_avgflx_em). The averaging period *T* is equal to the primary output interval of the WRF model (namelist variable history_interval) and set to 30 min for this study. The time average of a quantity $A=\bar{\tilde{a}}$ and a counter *n* are both set to zero at the start of an averaging interval *T*. At the end of every Runge–Kutta (R‐K) time step (*t*_1_), the average value is updated with the instantaneous value $\tilde{a}$ following

(B1) $$\begin{array}{cc} A\left(x,y,z,t_{1}\right) & =\bar{\tilde{a}}\left(x,y,z,t_{1}\right)\\ & =\frac{n\;A\left(x,y,z,t_{0}\right)+\tilde{a}\left(x,y,z,t_{1}\right)}{n+1}, \end{array}$$

with *t*_0_ representing the time at the end of the previous R‐K time step. The counter *n* is increased by one after updating *A*. This averaging procedure is an efficient way to keep memory allocations as low as possible during the real‐case WRF LES.

Time‐averages of the three wind components *U*, *V*, *W* and potential temperature Θ are calculated during the simulation, as well as individual products of these quantities. In a post‐processing step, the latter are used to derive the resolved (co‐)variances of these quantities for each averaging interval following

(B2) $$\begin{array}{cc} {\bar{u^{\prime 2}}}_{\text{RES}} & =\bar{\tilde{u}\tilde{u}}-U^{2},\;{\bar{v^{\prime 2}}}_{\text{RES}}=\bar{\tilde{v}\tilde{v}}-V^{2},\\ {\bar{w^{\prime 2}}}_{\text{RES}} & =\bar{\tilde{w}\tilde{w}}-W^{2}, \end{array}$$

and

(B3) $$\begin{array}{cc} {\bar{u^{\prime }\theta^{\prime }}}_{\text{RES}} & =\bar{\tilde{u}\tilde{\theta }}-U\Theta ,\;\;{\bar{v^{\prime }\theta^{\prime }}}_{\text{RES}}=\bar{\tilde{v}\tilde{\theta }}-V\Theta ,\\ {\bar{w^{\prime }\theta^{\prime }}}_{\text{RES}} & =\bar{\tilde{w}\tilde{\theta }}-W\Theta . \end{array}$$

Resolved velocity variances are also used to derive resolved TKE (Section 2.4). The SGS part to the velocity variances are calculated after Deardorff (1980) at every R‐K time step and averaged following Equation (B1) to be consistent with the resolved part. The same procedure is applied to derive averaged values for the SGS heat fluxes following Deardorff (1980).

In order to evaluate the different contributions to the CAP erosion, individual instantaneous contributions of Equation (2) are diagnosed during each R‐K time step and averaged over the interval *T* = 30 min. The change of potential temperature during a R‐K time step resulting from the radiation and microphysics parametrizations are readily available in WRF and time‐averaged following Equation (B1) to yield $\bar{S_{\text{RAD}}}$ and $\bar{S_{\text{MP}}}$, respectively, in Equation (2). The change of potential temperature due to SGS turbulent mixing during a R‐K time step is derived following Deardorff (1980) for all individual Cartesian components and averaged using Equation (B1) to yield

$$\begin{array}{ll} \bar{S_{\text{SGSTRB}}}=\bar{S_{\text{SGSTRBx}}}+\bar{S_{\text{SGSTRBy}}}+\bar{S_{\text{SGSTRBz}}} & \end{array}$$

in Equation (2).

Within the dynamical core, advection can be calculated in various ways based on namelist settings, with positive‐definite fifth (third) order as the default for horizontal (vertical) advection (Skamarock *et al*., 2019), which is also used in this study. Horizontal advection is calculated in the WRF model along terrain‐following model levels. In order to diagnose the change of potential temperature due to the Cartesian components of mean flow advection (MADV) and resolved turbulent mixing (RESTRB; Equation 3), a separate diagnostic tool is added to the WRF source code. The Cartesian components of advection are diagnosed based on first‐order differences in the physical space. Horizontal and vertical gradients are calculated based on previous work of Wagner *et al*. (2014) and Leukauf *et al*. (2015), and are adapted from a part of WRF standard code for the calculation of diffusion in the physical space in module_diffusion.F. During each R‐K time step, the instantaneous total advection of potential temperature is diagnosed from the sum of the three instantaneous Cartesian components. Additionally, each component is averaged over the interval *T* and these averages are summed to yield the averaged total advection

(B4) $$-\bar{\tilde{v}\cdot \nabla \tilde{\theta }}=-\bar{\tilde{u}\frac{\partial \tilde{\theta }}{\partial x}}-\bar{\tilde{v}\frac{\partial \tilde{\theta }}{\partial y}}-\bar{\tilde{w}\frac{\partial \tilde{\theta }}{\partial z}}$$

appearing in Equation (2). At the end of an averaging interval *T*, the three components of mean flow advection MADV are calculated by applying the same procedure on averaged quantities as illustrated in Equation (3). Finally, the three Cartesian components of RESTRB (Equation 3) are calculated offline in a post‐processing step as the difference between total and mean‐flow advection following

(B5) $$\begin{array}{c} \bar{u^{\prime }\frac{\partial \theta^{\prime }}{\partial x}}=\bar{\tilde{u}\frac{\partial \tilde{\theta }}{\partial x}}-U\frac{\partial \Theta }{\partial x},\;\bar{v^{\prime }\frac{\partial \theta^{\prime }}{\partial y}}=\bar{\tilde{v}\frac{\partial \tilde{\theta }}{\partial y}}-V\frac{\partial \Theta }{\partial y},\\ \bar{w^{\prime }\frac{\partial \theta^{\prime }}{\partial z}}=\bar{\tilde{W}\frac{\partial \tilde{\theta }}{\partial z}}-W\frac{\partial \Theta }{\partial z}. \end{array}$$

The numeric value of the total advection diagnosed by Equation (B4) is not exactly the same as the one calculated by the dynamical core of the WRF model. Nevertheless, with our approach all advective and turbulent heating tendencies are available as Cartesian components in physical space (which facilitates physical interpretation of CAP dynamics) and they are independent of the type of prognostic temperature variable used in the dynamical core (in our case a so‐called moist potential temperature, which is the default prognostic variable since WRF version 4.0), and the heat budget is closed due to the derivation of turbulent components as residuals (Equation B5). Beside the potential temperature tendency terms, the acceleration of the horizontal wind components $\tilde{u}$ and $\tilde{v}$ due to the pressure gradient force is diagnosed during each R‐K step of the simulation following the approach by Lehner (2012). Additionally, the instantaneous forcing terms are averaged during interval *T* using Equation (B1).

The modifications to the WRF source code also affect the extrapolation of the input dataset (in our case ECMWF analysis) to WRF model levels that lie below the lowest level of the input data during the execution of real.exe. The user can choose if temperature extrapolation results in isothermal conditions, a standard atmosphere lapse‐rate of 6.5 K·km^−1^ or neutral conditions via the namelist variable t_extrap_type. However, as the ECMWF analysis supplies thermodynamic temperature, these options only yield correct results if the namelist variable interp_theta is set equal to TRUE to calculate a potential temperature from the input data before extrapolation. An additional option (t_extrap_type = 4) has been added to specify a user‐defined temperature lapse rate in K·m^−1^ with the newly introduced namelist variable t_extrap_lr. MESO‐DEF uses t_extrap_type =2 (standard atmosphere lapse‐rate) and interp_theta = TRUE, resulting in a mean potential temperature bias of about 0.8 (1.1) K north (south) of the main Alpine crest at 1200 UTC on 3 November 2017 (marker in Figure A1b,c). Using a user‐specified lapse‐rate for extrapolation for MESO‐NUD (t_extrap_type =4, interp_theta = TRUE and t_extrap_lr = 0.0025 K·m^−1^ based on observations in the Inn Valley) reduces the mean bias to about –0.4 (0.1) K, (Figure A1b,c). Moreover, a namelist variable is introduced to let the user decide to use the default WRF formulation of the length‐scale $\ell =\sqrt[{3}]{\Delta x\Delta y\Delta z}$ or the formulation used in Schmidli (2013) of ℓ=Δx to avoid a change of the turbulent length‐scale due to a stretching in the vertical grid spacing Δz with increasing height in the WRF mode (Schmidli, 2013). The turbulent length‐scale *ℓ* is used in SGS turbulent mixing after Deardorff (1980) to calculate eddy diffusivities and eddy viscosities. However, the impact of these two different formulations of *ℓ* on the presented numerical simulations is very minor.

## POWER SPECTRAL DENSITY ANALYSIS

### 1

The ability of the LES to capture the dominant part of the turbulence is assessed by comparing the power spectral density (PSD) of the simulated wind of MESO‐NUD, LES‐dx200 and LES‐dx40 with observations from the EC station EC_W and the Doppler wind lidar SL74 (Figure 1c) in Figure C1. Temporal resolution of EC data is 20 Hz, while the SL74 measured at approximately 1 Hz. For this study, only vertical stare measurements of the SL74 during 18 min time windows once an hour are available. Identical time windows are used for the WRF data. For the comparison between EC and WRF data, 30 min time windows are used and a single rotation of the horizontal wind is performed for both datasets to align the *U*‐component with the mean wind direction. For the comparison between WRF and lidar data, no rotation is performed. WRF data from every integration time step at the nearest grid point to the respective observations is used to derive power spectral densities at a sampling frequency of 0.4 Hz for MESO‐NUD, 1.33 Hz for LES‐dx200, and 4 Hz for LES‐dx40. Individual PSDs are calculated using Welch's method on 18‐ and 30‐min Blackman–Harris time windows with no overlapping for SL74 and EC, respectively, and are normalized by the variance of the respective wind component during the time window. Model data and observations are linearly detrended for each individual time window. To gain more robust results, individual spectra are subsequently averaged over a 7.5 hr period from 2230 UTC to 0600 UTC on 4 November. Additionally, averaging over 80 equally‐sized frequency bins in the log_10_‐space is performed to remove noise, especially from the high‐frequency end of the spectrum. Sharp drops in the PSD for WRF model data are visible for frequencies higher than the effective model resolution of the corresponding simulation in Figure C1. This frequency threshold is indicated for LES‐dx40 by a vertical dashed line in Figure C1 at $\sqrt{U^{2}+V^{2}}$ (7Δx )^−1^ (with the mean simulated horizontal wind components *U* and *V* and the horizontal grid spacing Δx = 40 m) and is based on the effective *spatial* resolution of 7Δx of the WRF model (Skamarock, 2004). Note that the part of the PSD for MESO‐NUD above 2 × 10^−2^ Hz in Figure C1c is considered as noise.

The averaged observed PSD follows the ‐5/3 slope, characteristic for the inertial subrange (Wyngaard, 2010) for frequencies above 10^−2^ Hz in Figure C1a and 3 × 10^−2^ Hz in Figure C1b. LES‐dx40 is not able to explicitly resolve the inertial subrange characterized by the energy transport towards smaller eddies. In fact, LES‐dx40 even overestimates the energy‐containing eddies compared to observations in this frequency range and up to the effective model resolution at 900 m amsl in Figure C1a. As mentioned in Section 4.5, the wavelength of K‐H waves and the diameter of eddies resulting from breaking K‐H waves (cf. Figure 3g‐i) in the foehn–CAP interaction layer is too large in LES‐dx40 compared to lidar observations by Haid *et al*. (2020). This suggests that energy‐containing eddies in LES‐dx40 remain too big which in turn leads to partly too vigorous turbulent mixing in the model. These eddies are then mostly dissipated by numerical diffusion in the model. Moreover, differences in the observed and simulated flow regime contribute to differences of the normalized PSD at 900 m amsl. While the observations are taken within the foehn–CAP interaction zone, a stronger direct influence of foehn is present at this height due to a shallower and/or weaker CAP in the simulations (cf. Figure 8 for LES‐dx40 data). Figure C1c shows a comparison between the PSD of the horizontal wind component along the mean wind direction at EC_W observed at around 4 m agl and simulated at 10 m agl (lowest model level). The peak in the PSD at low frequencies is very well reproduced by LES‐dx40, while the inertial subrange at this location near the surface is well beyond the effective resolution of LES‐dx40.

Naturally, the MESO‐NUD simulation (Δx = 1 km) is not able to resolve the small‐scale vertical and horizontal motions and therefore the normalized PSD is strongly reduced for frequencies higher than about 2 × 10^−3^ s^−1^ compared to LES‐dx40 and observational data (Figure C1). In contrast, MESO‐NUD and LES‐dx200 overestimate the normalized PSD at the lowest frequencies shown in Figure C1a, b. LES‐dx200 is able to resolve the largest eddies at the low‐frequency range of the observed spectrum in Figure C1a, b. However, in contrast to LES‐dx40, it completely fails to capture even a small part of the inertial subrange. Interestingly, the PSDs of the wind component rotated towards the mean wind direction for the two LESs and the observations are very similar for low frequencies less than 2 × 10^−3^ Hz (Figure C1c). However, even LES‐dx40 is not able to capture the inertial subrange at the surface. This is no surprise since the lowest part of the CAP is expected to be characterized by small‐scale turbulence that cannot be resolved with a model mesh size of Δx = 40 m. Here, the model mostly relies on the effect of SGS mixing (cf. Figure 9).
